# Evaluating the anti-gut dysbiotic potential of bioactive primary metabolites derivatives from *Lactaplantibacillus plantarum* 12–3. An integrated ADMET, network pharmacology, molecular docking and normal mode analysis approaches

**DOI:** 10.3389/fnut.2026.1844467

**Published:** 2026-06-12

**Authors:** Tariq Aziz, Emmanuel Pascal Owona, Nadège Thérèse Amougou Okoa, Mengue Ngadena Yolande Sandrine, Inam Ur Rahman, Abid Sarwar, Liqing Zhao, Zhennai Yang, Metab Alharbi, Abdullah F. Alasmari

**Affiliations:** 1 State Key Laboratory of Intelligent Construction and Healthy Operation and Maintenance of Deep Underground Engineering, Shenzhen University, Shenzhen, China; 2 School of Biomedical Engineering Shenzhen University, Shenzhen, Guangdong, China; 3 Key Laboratory of Geriatric Nutrition and Health of Ministry of Education, Beijing Advanced Innovation Center for Food Nutrition and Human Health, Beijing Engineering and Technology Research Center of Food Additives, Beijing Technology and Business University, Beijing, China; 4 Department of Animal Biology and Physiology, Laboratory of Animal Physiology, Faculty of Science, University of Yaoundé 1, Yaoundé, Cameroon; 5 Department of Animal Biology and Physiology, Laboratory of Environment and Hydrobiology, Faculty of Science, University of Yaoundé 1, Yaoundé, Cameroon; 6 Fuli Institute of Food Science, College of Biosystems Engineering and Food Science, Zhejiang University Hangzhou, Hangzhou, China; 7 Department of Pharmacology and Toxicology, College of Pharmacy, King Saud University Riyadh, Riyadh, Saudi Arabia

**Keywords:** ADMET analysis, BRAF, dynamic simulation, intestinal dysbiosis, *Lactaplantibacillus plantarum*, molecular docking

## Abstract

**Introduction:**

Intestinal dysbiosis is a disorder of the gut microbiome, characterized by a loss of equilibrium between the microorganisms found in the gastrointestinal tract and their hosts. It leads to metabolic changes in both the gut and in the body’s inflammatory response, and adversely affects the epithelial cells that line the intestines. This article aimed to study the mechanisms whereby three metabolites, produced from *Lactiplantibacillus plantarum*, including 2,4-decadienal, (Z)-ethyl heptadec-9-enoate, and octadecanoic acid, may alter protein activity associated with diseases of the gut.

**Methods:**

Using a variety of in-silico methods, including ADMET modeling and prediction, docking of two target proteins, Fatty Acid–Binding Protein 4 (FABP4) and B-Raf Proto-Oncogene, Serine/Threonine Kinase (BRAF), and molecular normal mode analysis and network pharmacology, we investigated the potential interaction of these compounds. Dynamics simulation was performed using GROMACS (2019.2) and GROMOS96 (43a1) force fields.

**Results and discussion:**

ADMET indicates good oral absorption, moderate ability to dissolve in your intestinal tract (lipophilicity), and low toxicity when consumed. Additionally, molecular docking techniques indicated that metabolite-protein binding is stable via primarily hydrophobic bonds, hydrogen bonds, and all have similar binding energies in the range of −5.1 to −6.2 kcal/mol. Normal mode analysis and dynamic simulation confirmed that the metabolite-protein complexes were stable. Network pharmacology studies suggest that the use of *L. plantarum*-derived metabolites as BRAF and FABP4 regulators of dysbiosis in the gut may result in therapeutic targets to restore homeostasis in the epithelial lining of the intestines and reduce inflammation.

**Conclusion:**

This work demonstrates the protective potential against intestinal dysbiosis of primary metabolites of *L. plantarum*. However, future experiments are essential to verify the predictions made from the in silico studies and optimize various types of metabolite-based treatments.

## 1 Introduction

Gut dysbiosis has been defined as an alteration of the gut microbiome designation, where there is decreased microbial diversity and an unfavorable ratio of beneficial vs. pathogenic microorganisms, resulting in altered metabolism, compromised gut barrier function, and chronic low-grade inflammation (1). It is increasingly recognized as a primary contributing factor to many types of gastrointestinal and extraintestinal diseases globally (2). The World Health Organization has not yet established a specific category for the disease of dysbiosis; however, estimates indicate that between 3 to 5% of the worldwide population is affected by functional or organic diseases associated with dysbiosis (2, 3). Dysbiosis is now seen in most people suffering from Inflammatory Bowel Disease (IBD), a group of chronic inflammatory diseases that are known to have a drastically increased global prevalence, from approximately 396/100,000 in 1990 to greater than 523/100,000 at present (4). This trend corresponds with changes in lifestyle, urbanization, and increased use of antibiotics (5, 6). In Asia, the impact of microbiota-related bowel disease is large. Estimates of IBS prevalence within some cohorts in South Asia have been reported to range from approximately 9.5 to 26%, including in Pakistan (7). Various studies conducted in Africa among diverse populations show similarly high rates of prevalence for functional bowel disorders (usually caused by dysbiosis) that exist due to inadequate sanitation, nutrition, and access to healthcare (8, 9). Dysbiosis has many clinical symptoms including abdominal discomfort, changes in bowel patterns (either the presence or absence of diarrhea), bloating, malabsorption, fatigue, and impaired immune function which when experienced long-term can lead to chronic disease, including inflammatory bowel disease, metabolic syndrome, colon cancer and other systemic inflammatory diseases (10, 11).

The mechanisms at a molecular level by which gastrointestinal dysbiosis occurs are dependent on numerous complex interactions between the host and the gut microbiota that negatively impact both the integrity of the intestinal barrier as well as immune system homeostasis and cellular signaling pathways (1). Dysbiosis causes an increase in the permeability of the intestines, resulting in the activation of pro-inflammatory signaling pathways initiated by microbial metabolites for example, lipopolysaccharides (LPS) (12). BRAF, one of the serine/threonine kinases signaling via the MAPK pathway, regulates epithelial cell proliferation, differentiation, and the ability to respond to inflammatory stimuli. Study findings have shown that aberrant BRAF activity is associated with intestinal inflammation, colorectal cancer development, and microbiota-mediated epithelial dysfunction (13, 14). Similarly, FABP4 (Fatty Acid Binding Protein 4), a lipid chaperone involved in transporting fatty acids and in relaying cellular metabolic information, is over-expressed in individuals with dysregulated lipid metabolism, chronic inflammatory conditions, and who are insulin-resistant. Thus, both proteins represent integrated molecular centers linking microbial dysbiosis to the metabolic and inflammatory dysfunctions in the gut and therefore represent promising targets for pharmacotherapy (15, 16). The current treatments for disabling gut bacteria imbalance include food, medicine, and hygiene (17, 18). Medications are the dominating choice of treatment, including antibiotics, anti-inflammatories, pre and probiotics, and on rare occasions a fecal implant (18). Unfortunately, antibiotics tend to disrupt the balance of the gut microbiota, promote the emergence of antibiotic-resistant bacteria, and cause undesirable side effects such as diarrhea and an overgrowth of the body’s natural bacteria, thereby creating conditions that weaken the immune system (19, 20). Probiotics and prebiotics’ effectiveness is inconsistent from strain to strain, host to host (21, 22). While improving sanitation and controlling infections can stop harmful gut bacteria from getting out of hand, they are not effective at correcting the damage already caused by a gut bacteria imbalance once the imbalance is present (1, 23). Although eating foods high in fiber, polyphenol-rich foods, and reducing consumption of ultra-processed foods may partially restore a healthy level of gut bacteria in a patient, these foods are challenging to continue for some patients (24, 25). All these treatments are limited by their ability to have inconsistent results, unwanted side effects, and our incomplete understanding of how human gut bacteria interact with our bodies’ molecularly (26).

The rise of interest in the role of bacteria as potential renewable resources of bioactive chemicals that can regulate the non-homogeneous gut microbiome may be attributed to the establishment of bacteria as a premier source of bioactive chemicals. Bacteria produce a variety of bioactive compounds that can alter inflammatory responses, help prevent oxidative damage to the host, and have some immunomodulatory properties. Bacterial metabolites, including short- and long-chain fatty acids, aldehydes, fatty acid esters, and antibiotics, can alter inflammation and oxidative stress in the human body by interacting with various host molecular receptors and pathways associated with dysbiosis or dysregulated gut health. Recent advancements and innovations in the computational identification and mechanistic evaluation of microbial bioactive compounds through ADMET profiling, pharmaconal networks, molecular docking, and molecular Normal mode analysis will potentially catalyze the development of new microbiota-derived therapeutics with enhanced safety and effectiveness profiles.

*Lactaplantibacillus plantarum* is an established lactic acid bacterium that can be found in fermented foods and has developed in the human gastrointestinal tract (27). This bacterium has shown the potential for many different probiotic benefits, which include: inhibition of pathogen growth, increasing intestinal barrier function, modulating immune response, and regulating lipid and glucose metabolism (28). Numerous studies have demonstrated that this bacterium has the ability to treat gastrointestinal disorders, metabolic syndrome, inflammatory diseases, and conditions associated with oxidative stress (29, 30). *L. plantarum* also produces fatty acids and volatile molecules that have bioactive properties, which can include: (1) octadecanoic acid, (2) 2,4-decadienal, and (3) (Z)-ethyl heptadec-9-enoate; it is possible these products can restore microbial and molecular homeostasis. No study to date has demonstrated the effects of these ubiquitous primary compounds in several food sources against intestinal dysbiosis, either through a computational approach or experimental validation. The purpose of the present study is to investigate the anti-dysbiotic mechanisms of these bioproducts using an integrated in silico approach involving (1) ADMET analysis, (2) network pharmacology, (3) molecular docking, and (4) molecular Normal mode analysis. This manuscript will introduce new data that demonstrates the ability of *L. plantarum*-derived fatty acid metabolites to produce multiple-target molecular interactions against known intestinal dysbiosis-related signaling pathways; therefore, they potentially will be the subject of a new class of microbiome-based therapeutics.

## 2 Materials and methods

### 2.1 Materials

#### 2.1.1 Bioactive compounds

**Figure 1:**
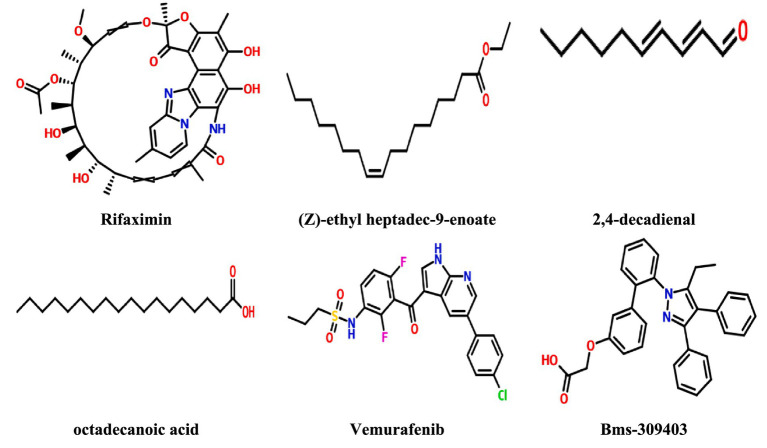
Compound from *L. plantarum* and reference drugs.

This research focused on three metabolites from *Lactaplantibacillus plantarum,* including octadecanoic acid, 2,4-decadienal, and (Z)-ethyl heptadec-9-enoate. Their chemical structures were retrieved in Structure Data File (SDF) format from the PubChem database^1^ (Figure 1).

#### 2.1.2 Target proteins

Two target proteins have been used in this study, including BRAF (B-Raf proto-oncogene serine/threonine kinase), and FABP4 (Fatty Acid Binding Protein 4). Table 1 shows the characteristics of these proteins.

**Table 1:** List of protein targets and binding site parameters.

ID name	PDB ID	Organism	Resolution	Binding site center		
				X	Y	Z
FABP4	1TOU	*Homo sapiens*	2.000	21.9549	4.7168	2.1007
BRAF	1UWH	*Homo sapiens*	2.950	95.3323	29.5049	69.1218

#### 2.1.3 Databases, software, and online platforms

The bioinformatic tools used in this study include PubChem, SwissADME, Swiss Target Prediction, Gene Cards, STRING, Cytoscape, Metascape, 1-Click Docking platform, BIOVIA Discovery Studio Visualizer, Imods, PLIP, and the SAVES v6.1 structure validation server.

### 2.2 Methods

#### 2.2.1 GCMS analysis

To perform GCMS analysis of *Lactaplantibacillus plantarum*, *L. plantarum* was isolated, and its metabolites were extracted. *L. plantarum* 12–3 was isolated from Tibetan kefir and maintained at the culture collection of the Dairy Laboratory of the Beijing Technology and Business University, and the metabolite was isolated according to Aziz et al. (31) and (32). The Shimadzu (2010) instrument coupled with a dual-stage TMP (Ultra) mass spectrometer was used to analyze the prepared fatty acid methyl esters (FAME) sample. 2 μL of the FAME sample was injected in a split mode (10:1) at 250 °C. Helium was used as a carrier gas with a flow rate of 1 mL/min. The fatty acid metabolites were separated on a highly polar fused silica capillary column (TR, Wax MS, 30 m length, 0.25 mm i.d, 0.25 m thickness) (Thermo Fisher Scientific). The oven temperature condition was initially set at 170 °C for 1 min, then ramped at 0.8 °C/min to 200 °C. The temperature of the ion source and line transfer were adjusted to 200 °C and 250 °C, respectively. The MS detector was operated in an electron ionization (EI) voltage of 70 eV under a mass scan range of 33–450 amu (m/z).

#### 2.2.2 ADMET prediction and drug-likeness assessment

The pharmacokinetic/toxicological profiles of the selected compounds were predicted using the SwissADME web service^2^ in terms of absorption, distribution, metabolism, and excretion attributes such as gastrointestinal absorption, blood–brain barrier permeability, P-glycoprotein interaction, and inhibitory effects on cytochrome P450 enzymes (33, 34).

#### 2.2.3 Network pharmacology analysis

##### 2.2.3.1 Target prediction and disease gene collection

The identified molecular targets of the assessed compounds were hypothesized using Swiss Target Prediction.^3^ Genes related to intestinal dysbiosis, gut inflammation, metabolic/inflammation, and microbiota imbalances were identified using the GeneCards database.^4^ A Venn diagram of these two datasets was constructed in Interactivenn webserver.^5^

##### 2.2.3.2 Protein–protein interaction network construction

The overlapping targets were then uploaded onto the STRING database^6^ to generate a protein–protein interaction (PPI) network, where the reference organism selected was *Homo sapiens*. The PPI network was then visualized using the software tool Cytoscape, where key topological parameters were calculated to analyze which proteins are potential hubs in the modulation of disease (34–36).

##### 2.2.3.3 Functional enrichment analysis

Functional enrichment analyses, including Gene Ontology (GO) analyses, were performed using the Metascape platform.^7^ Enriched biological processes were identified based on statistically significant thresholds and constructed in SRplot server^8^ (34, 37).

#### 2.2.4 Validation of protein structures

Structural validation of target proteins was done using the SAVES v6.1 online server^9^ for the selected proteins, such as BRAF and FABP4. For this, the Ramachandran plot analysis was done, which provides information on the stereochemical quality of protein structures based on the distribution of the *φ* (phi) and *ψ* (psi) torsion angles. A threshold of ≥ 90% of residues in the favored regions of the Ramachandran plot defined the structurally reliable proteins for the docking process, and those proteins were selected for the study.

#### 2.2.5 Molecular docking analysis

##### 2.2.5.1 Preparation of ligands and proteins

Protein structures that were checked for correctness via SAVES v6.1^10^ were made by initially removing water molecules and non-structural heteroatoms. Later on, hydrogens were added, and the structures were subjected to energy minimization. Ligand structures were subjected to energy minimization and subsequently converted into compatible formats for docking simulations.

##### 2.2.5.2 Docking execution

Molecular docking experiments were performed on the 1-Click Docking platform,^11^ which allows for the automation of active site detection and ligand-protein interaction modeling. The ligands were docked one by one with the experimentally confirmed target proteins, and binding affinity scores were calculated (38).

##### 2.2.5.3 Visualization and interaction profiling

Docked complexes were visualized using BIOVIA Discovery Studio Visualizer to analyze hydrogen bonds, hydrophobic interactions, electrostatic forces, and other key molecular interactions contributing to binding stability (35, 38).

#### 2.2.6 Normal mode analysis

The interactions of ligand protein complexes with stability, flexibility, and conformational changes were determined using the iMODS webserver.^12^ Normal mode analysis was carried out to determine the deformability, eigenvalues, covariance matrices, and elastic network models (36).

#### 2.2.7 Pharmacophore analysis

Pharmacophore modeling and interaction profiling using the PLIP (ProteinLigand Interaction Profiler) tool^13^ helped us to understand the interactions and binding patterns of the biological molecules. We identified the main pharmacophoric features determining ligand binding and biological activity and compared these features among different complexes (36).

#### 2.2.8 Dynamic simulation

A 100 ns molecular dynamics (MD) simulation on BRAF’s ligand-bound complexes was performed for the purpose of additional analysis using GROMACS (2019.2) and GROMOS96 (43a1) force fields (39–41). Files defining the proteins’ and ligands’ topology were generated using Charmm GUI (42, 43). The MD simulation had a solvation system and used periodic boundary conditions and simulated physiological conditions (44–46) to perform system minimization and equilibration under constant numbers of atoms, constant pressure (N), and constant temperature (T). The temperature (310 K) and pressure (1 atm) were maintained by using a velocity rescaling and Parrinello-Rahman barostat. A leap-frog integrator with a 2-fs time step was used to conduct the simulations, and each system’s MD had a duration of 100 ns with 1,000 total frames (0.1 ns per frame snapshot) taken from the MD trajectories to compute the RMSD, RMSF, ROG, SASA, and hydrogen bonds.

## 3 Results

### 3.1 GCMS analysis of *Lactaplantibacillus plantarum*

Table 2 presents the GC–MS profile of *Lactaplantibacillus plantarum*, revealing a complex mixture of organic acids, aldehydes, phenolic compounds, and fatty acid derivatives. The chromatogram is dominated by 9,12-octadecadienoic acid, methyl ester (43.10%) and trans,trans-9,12-octadecadienoic acid (32.30%), indicating a strong prevalence of unsaturated fatty acid–related compounds, followed by octadecanoic acid (12.96%). Minor constituents such as acetic acid (2.52%), (Z)-ethyl heptadec-9-enoate (2.15%), and phenol, 3,5-bis(1,1-dimethylethyl)- (1.07%).

**Table 2:** GCMS profile of *Lactaplantibacillus plantarum.*

Name	Peak#	R. Time	I. Time	F. Time	Area	Area %	Height	Height %	A/H
Acetic acid	1	5.889	5.840	6.010	299,466	2.52	61,237	3.66	4.89
2,4-Decadienal, (E, E)-	2	8.940	8.890	8.985	86,378	0.73	35,548	2.12	2.43
2,4-Decadienal	3	9.341	9.300	9.405	143,912	1.21	59,417	3.55	2.42
Octanoic acid	4	11.176	11.120	11.255	90,273	0.76	25,443	1.52	3.55
Dodecanamide, N-[4-(4-aminophenyl)s…]	5	11.589	11.455	11.610	39,104	0.33	4,663	0.28	8.39
Phenol, 3,5-bis(1,1-dimethylethyl)-	6	13.159	13.115	13.235	126,979	1.07	41,518	2.48	3.06
1,2-Ethanediamine, N, N′-dibutyl-	7	13.955	13.885	14.020	72,144	0.61	17,488	1.04	4.13
(Z)-Ethyl heptadec-9-enoate	8	15.260	15.190	15.340	255,371	2.15	66,599	3.98	3.83
(9Z,12Z,15Z)-1,3-Dimethoxypropan-2-y…	9	15.653	15.595	15.705	40,281	0.34	11,281	0.67	3.57
trans,trans-9,12-Octadecadienoic acid	10	15.885	15.800	16.015	3,831,984	32.30	918,168	54.80	4.17
Octadecanoic acid	11	25.416	25.250	25.685	1,538,123	12.96	148,001	8.83	10.39
(Z)-1,3-Dimethoxypropan-2-yl tetracos-	12	30.247	30.110	30.425	226,399	1.91	22,312	1.33	10.15
9,12-Octadecadienoic acid, methyl ester	13	32.608	32.320	33.240	5,113,777	43.10	263,705	15.74	19.39

### 3.2 ADMET profile of 2,4-decadienal, (Z)-ethyl heptadec-9-enoate

**Figure 2:**
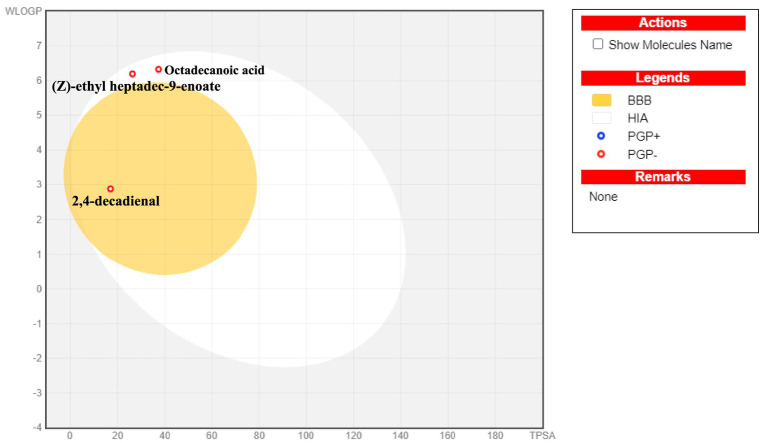
WLOGP–TPSA distribution and predicted blood–brain barrier permeability of the studied molecules.

Table 3 and Figure 2 depict the ADMET profile of 2,4-decadienal, (Z)-ethyl heptadec-9-enoate. 2,4-decadienal showed the lowest molecular weight (152.23 g/mol) among the three compounds, together with the smallest number of heavy atoms and a reduced number of rotatable bonds, which points to a less extended and less conformationally flexible structure. On the other hand, (Z)-ethyl heptadec-9-enoate and octadecanoic acid exhibited higher molecular weights (296.49 g/mol), and (284.48 g/mol) respectively, molecular flexibility, and structural complexity, features that might facilitate their binding to lipid, binding proteins. Regarding polarity and hydrogen bonding capacity, all compounds showed low topological polar surface area (TPSA < 30) and did not have hydrogen bond donors (except for octadecanoic acid), thus implying good membrane permeability. Nevertheless, 2,4-decadienal, which had only one hydrogen bond acceptor and a smaller TPSA value, presented a physicochemical profile in line with fast diffusion through biological membranes, including the blood–brain barrier. Meanwhile, the fatty acid derivatives had slightly higher TPSA values and hydrogen bond acceptor numbers, which could be favorable for the selective distribution of the intestine and peripheral tissues. Lipophilicity profiles revealed a clear differentiation between the compounds. 2,4-decadienal exhibited a moderate level of lipophilicity (consensus LogP = 2.85) and (consensus LogP = 5.93), respectively, hence it balanced its membrane permeability with its aqueous solubility. The other way around, (Z)-ethyl heptadec-9-enoate showed a very high level of lipophilicity (consensus LogP = 6.01). The same tendency was observed with the solubility experiments, where 2,4-decadienal was at all times considered a soluble compound, but the two compounds with the fatty acid long-chain structure showed a moderate to poor aqueous solubility depending on the model used for the prediction.

**Table 3:** ADMET profile of 2,4-decadienal, (Z)-ethyl heptadec-9-enoate, and Octadecanoic acid.

Parameters	2,4-decadienal	(Z)-ethyl heptadec-9-enoate	Octadecanoic acid
Physicochemical properties			
Formula	C10H16O	C19H36O2	C18H36O2
Molecular weight	152.23 g/mol	296.49 g/mol	284.48 g/mol
Num. heavy atoms	11	21	20
Num. arom. Heavy atoms	0	0	0
Fraction Csp3	0.50	0.84	0.94
Num. rotatable bonds	6	16	16
Num. H-bond acceptors	1	2	2
Num. H-bond donors	0	0	1
Molar Refractivity	49.44	94.26	90.41
TPSA	17.07 Å^2^	26.30 Å^2^	37.30 Å^2^
Lipophilicity			
Log Po/w (iLOGP)	2.67	5.05	4.30
Log Po/w (XLOGP3)	3.25	7.49	8.23
Log Po/w (WLOGP)	2.88	6.20	6.33
Log Po/w (MLOGP)	2.49	4.80	4.67
Log Po/w (SILICOS-IT)	2.96	6.54	6.13
Consensus Log Po/w	2.85	6.01	5.93
Water solubility			
Log S (ESOL)	−2.44	−5.34	−5.73
Solubility	5.59e-01 mg/mL; 3.67e-03 moL/L	1.35e-03 mg/mL; 4.56e-06 moL/L	5.26e-04 mg/mL; 1/85e-06 mol/l
Class	Soluble	Moderately soluble	Moderately soluble
Log S (Ali)	−3.28	−7.88	−8.87
Solubility	7.95e-02 mg/mL; 5.22e-04 moL/L	3.95e-06 mg/mL; 1.33e-08 moL/L	3.80e-07 mg/mL; 1.33e-09 moL/L
Class	Soluble	Poorly soluble	Poorly soluble
Log S (SILICOS-IT)	−2.00	−6.09	−6.11
Solubility	1.53e+00 mg/mL; 1.01e-02 moL/L	2.40e-04 mg/mL; 8.11e-07 moL/L	2.19e-04 mg/mL; 7.71–07 mol/l
Class	Soluble	Poorly soluble	Poorly soluble
Pharmacokinetics			
GI absorption	High	High	High
BBB permeant	Yes	No	No
P-gp substrate	No	No	No
CYP1A2 inhibitor	No	Yes	Yes
CYP2C19 inhibitor	No	No	No
CYP2C9 inhibitor	No	No	No
CYP2D6 inhibitor	No	No	No
CYP3A4 inhibitor	No	No	No
Log Kp (skin permeation)	−4.92 cm/s	−2.79 cm/s	−2.19 cm/s

With regards to pharmacokinetics, all three compounds demonstrated high predicted gastrointestinal absorption, which is in line with their use for oral administration and gut-directed therapeutic strategies. It is worth noting that 2,4-decadienal was predicted to be able to cross the blood–brain barrier, while (Z)-ethyl heptadec-9-enoate and octadecanoic acid were predicted not to; therefore, the latter two compounds are less likely to be exposed to the central nervous system. On the topic of transporter and metabolic interactions, the compounds were not predicted to be substrates of P-glycoprotein, suggesting less efflux and possibly higher intestinal retention. Yet, (Z)-ethyl heptadec-9-enoate and octadecanoic acid were found to be selective inhibitors of CYP1A2, whereas 2,4-decadienal was not predicted to inhibit any major cytochrome P450 isoforms. Predictions of skin permeation further set these chemicals apart. 2,4-decadienal showed lower skin permeation values, which is in line with its smaller size and moderate lipophilicity, whereas the fatty acid derivatives displayed higher predicted skin permeability.

Figure 2 reveals the location of the studied molecules on the WLOGP vs. TPSA chemical space. This space is widely used to evaluate oral absorption and blood–brain barrier (BBB) permeability. The yellow area denotes the best physicochemical space for the BBB penetration, whereas the bigger white area corresponds to the human intestinal absorption (HIA) friendly conditions. 2,4-decadienal is deep inside the BBB permeable area, with its lipophilicity being moderate (WLOGP 2.8) and very low polar surface area (TPSA 15), thus indicating a high probability of brain exposure. (Z)-ethyl heptadec-9-enoate and Octadecanoic acid, while still inside the BBB border, exhibit significantly higher lipophilicity (WLOGP 6.2) that may lead to higher non-specific binding and, hence, influence the pharmacokinetic profile. In addition, both chemicals are identified as the P-glycoprotein (PGP) non-substrates, that is, the limited efflux at the BBB is presumed.

### 3.3 Network pharmacology results

#### 3.3.1 Venn diagram

**Figure 3:**
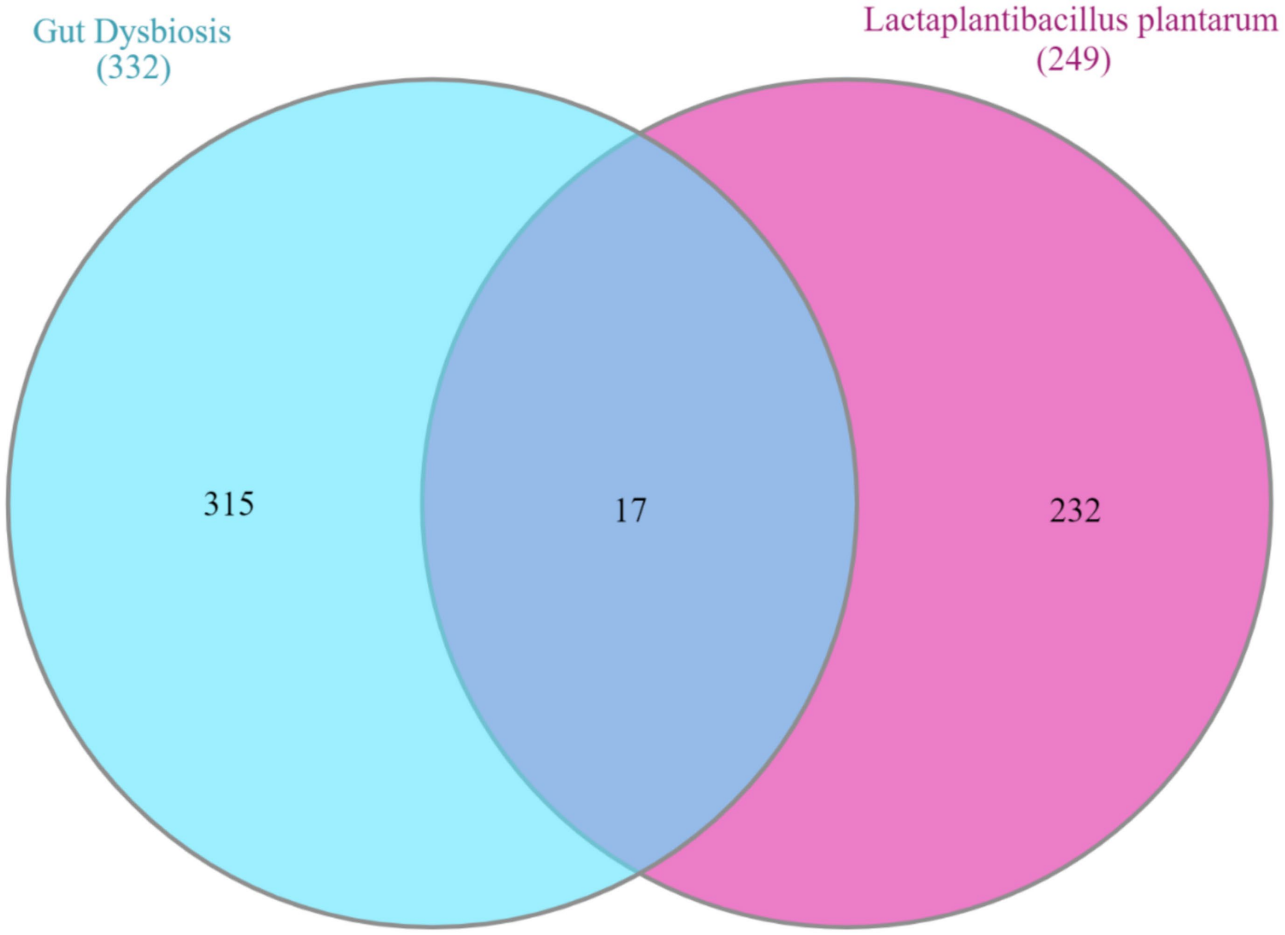
Venn diagram of gut dysbiosis genes and target proteins of *Lactaplantibacillus plantarum* compounds.

In order to pinpoint the possible molecular targets that *Lactaplantibacillus plantarum*-derived metabolites could use for their anti-dysbiotic properties, a comparative target analysis was conducted between the genes associated with gut dysbiosis and the targets related to *Lactaplantibacillus plantarum*. As shown in Figure 3, initially, a sum of 332 genes associated with dysbiosis and 249 targets related to *L. plantarum* were collected from publicly available databases. The Venn diagram analysis demonstrated the presence of 17 overlapping targets, which made up the shared molecular intersection of gut dysbiosis and *L. plantarum*.

#### 3.3.2 Protein–protein interaction

**Figure 4:**
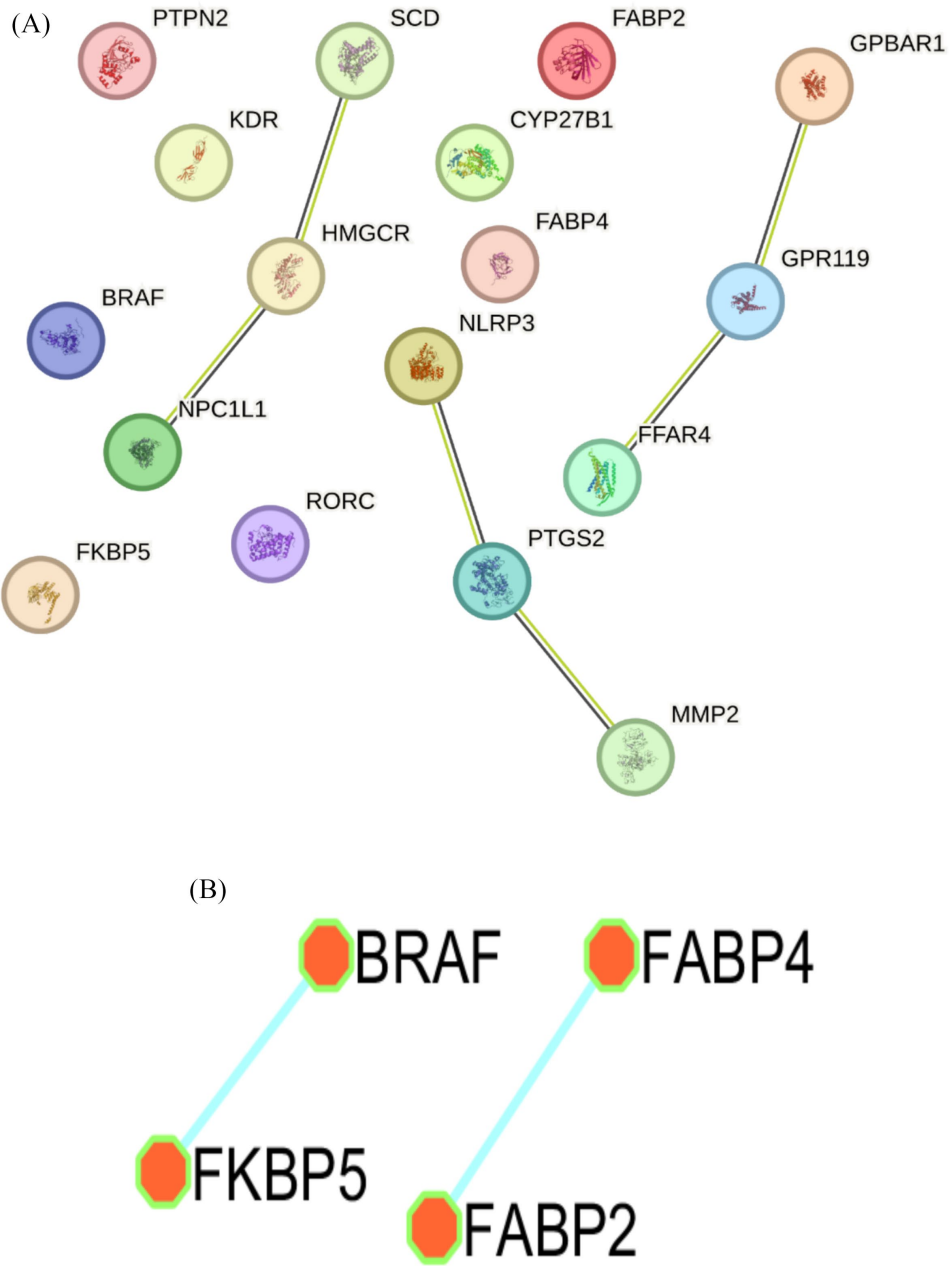
**(A)** Protein–protein interaction constructed in STRING database. **(B)** Protein–protein interaction constructed in Cytoscape.

Figures 4A,B and Table 4 show that *Lactaplantibacillus plantarum* compounds can bind to BRAF, FABP4, FABP2, and FKBP5 proteins. FABP2 and FKBP5 are network-identified targets, but we have not validated these proteins for docking simulation.

**Table 4:** Top 4 of target proteins of *L. plantarum* metabolites involved in gut dysbiosis.

Rank	Gene ID	Names	Degree	Betweenness	Closeness
1	673	BRAF	Degree: 2.0	Betweenness: 0.23746	Closeness: 0.33333334
2	2,167	FABP4	Degree: 2.0	Betweenness: 0.23746	Closeness: 0.33333334
3	2,169	FABP2	Degree: 2.0	Betweenness: 0.23746	Closeness: 0.33333334
4	2,289	FKBP5	Degree: 2.0	Betweenness: 0.23746	Closeness: 0.33333334

#### 3.3.3 Compound-target network

**Figure 5:**
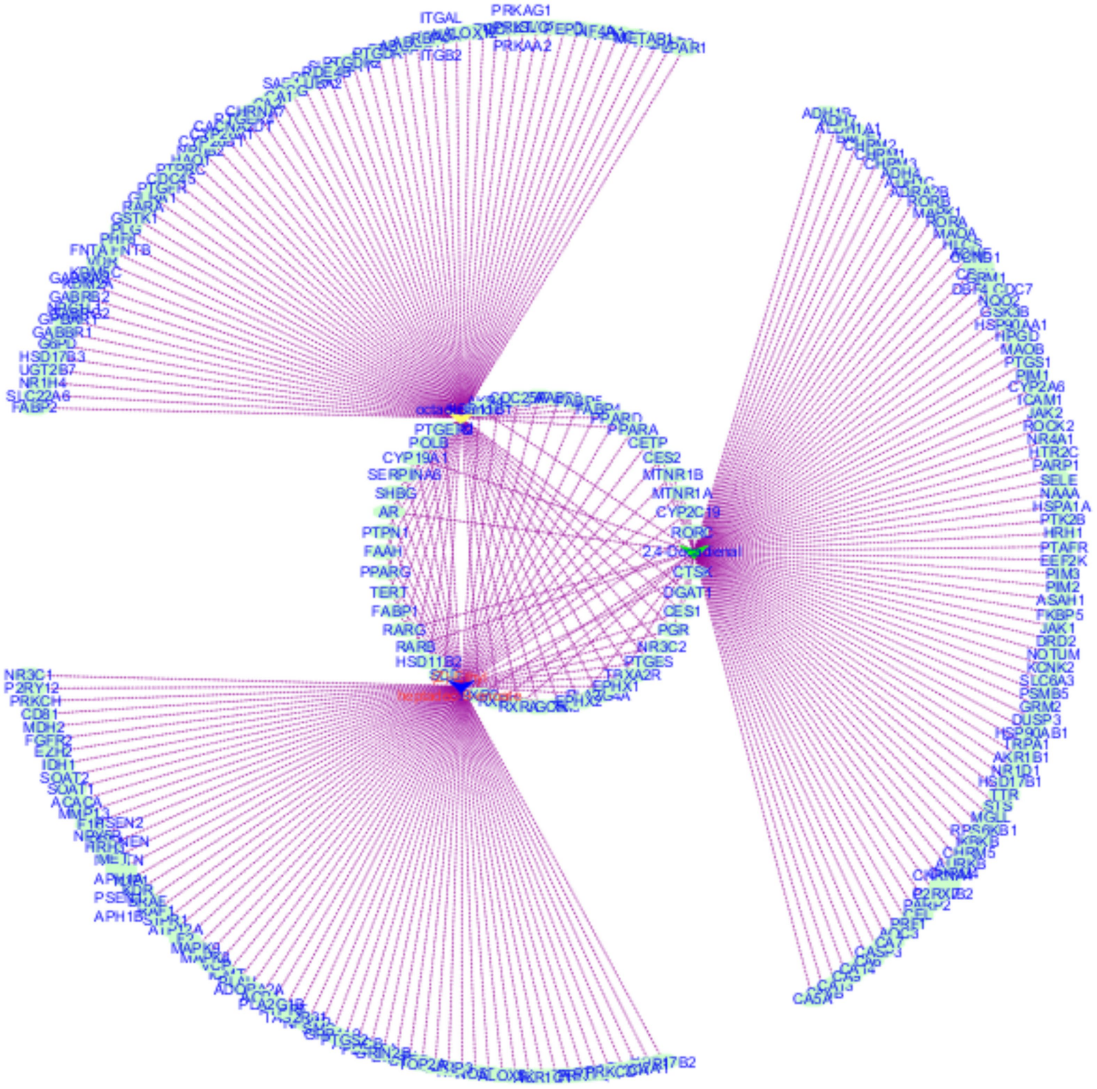
Compound target network constructed in Cytoscape.

Table 5 and Figure 5 present the protein targets of *L. plantarum* metabolites. These proteins include EPHX1 (rank 1), CES1 (rank 2), AR (rank 3), HSD11B1 (rank 4), PGR (rank 5), and CYP19A1 (rank 6), centrality values (betweenness = 2194.71; closeness = 0.503). We have also NR3C2 (rank 7), PTGES (rank 8), TBXA2R (rank 9), PLA2G4A (rank 10), GRM5 (rank 11), HMGCR (rank 12), SCD (rank 13), HSD11B2 (rank 14), FABP1 (rank 15), TERT (rank 16), PPARG (rank 17), FAAH (rank 18), and PTPN1 (rank 19) with lower but still considerable centrality (betweenness 296.16; closeness 0.381).

**Table 5:** Top 19 target proteins of compounds.

Rank	Genes	Degree	Betweenness	Closeness
1	EPHX1	Degree: 3.0	Betweenness: 2194.7078	Closeness: 0.5030181
2	CES1	Degree: 3.0	Betweenness: 2194.7078	Closeness: 0.5030181
3	AR	Degree: 3.0	Betweenness: 2194.7078	Closeness: 0.5030181
4	HSD11B1	Degree: 3.0	Betweenness: 2194.7078	Closeness: 0.5030181
5	PGR	Degree: 3.0	Betweenness: 2194.7078	Closeness: 0.5030181
6	CYP19A1	Degree: 3.0	Betweenness: 2194.7078	Closeness: 0.5030181
7	NR3C2	Degree: 2.0	Betweenness: 296.15543	Closeness: 0.3805175
8	PTGES	Degree: 2.0	Betweenness: 296.15543	Closeness: 0.3805175
9	TBXA2R	Degree: 2.0	Betweenness: 296.15543	Closeness: 0.3805175
10	PLA2G4A	Degree: 2.0	Betweenness: 296.15543	Closeness: 0.3805175
11	GRM5	Degree: 2.0	Betweenness: 296.15543	Closeness: 0.3805175
12	HMGCR	Degree: 2.0	Betweenness: 296.15543	Closeness: 0.3805175
13	SCD	Degree: 2.0	Betweenness: 296.15543	Closeness: 0.3805175
14	HSD11B2	Degree: 2.0	Betweenness: 296.15543	Closeness: 0.3805175
15	FABP1	Degree: 2.0	Betweenness: 296.15543	Closeness: 0.3805175
16	TERT	Degree: 2.0	Betweenness: 296.15543	Closeness: 0.3805175
17	PPARG	Degree: 2.0	Betweenness: 296.15543	Closeness: 0.3805175
18	FAAH	Degree: 2.0	Betweenness: 296.15543	Closeness: 0.3805175
19	PTPN1	Degree: 2.0	Betweenness: 296.15543	Closeness: 0.380517

#### 3.3.4 Biological processes involved in gut dysbiosis

**Figure 6:**
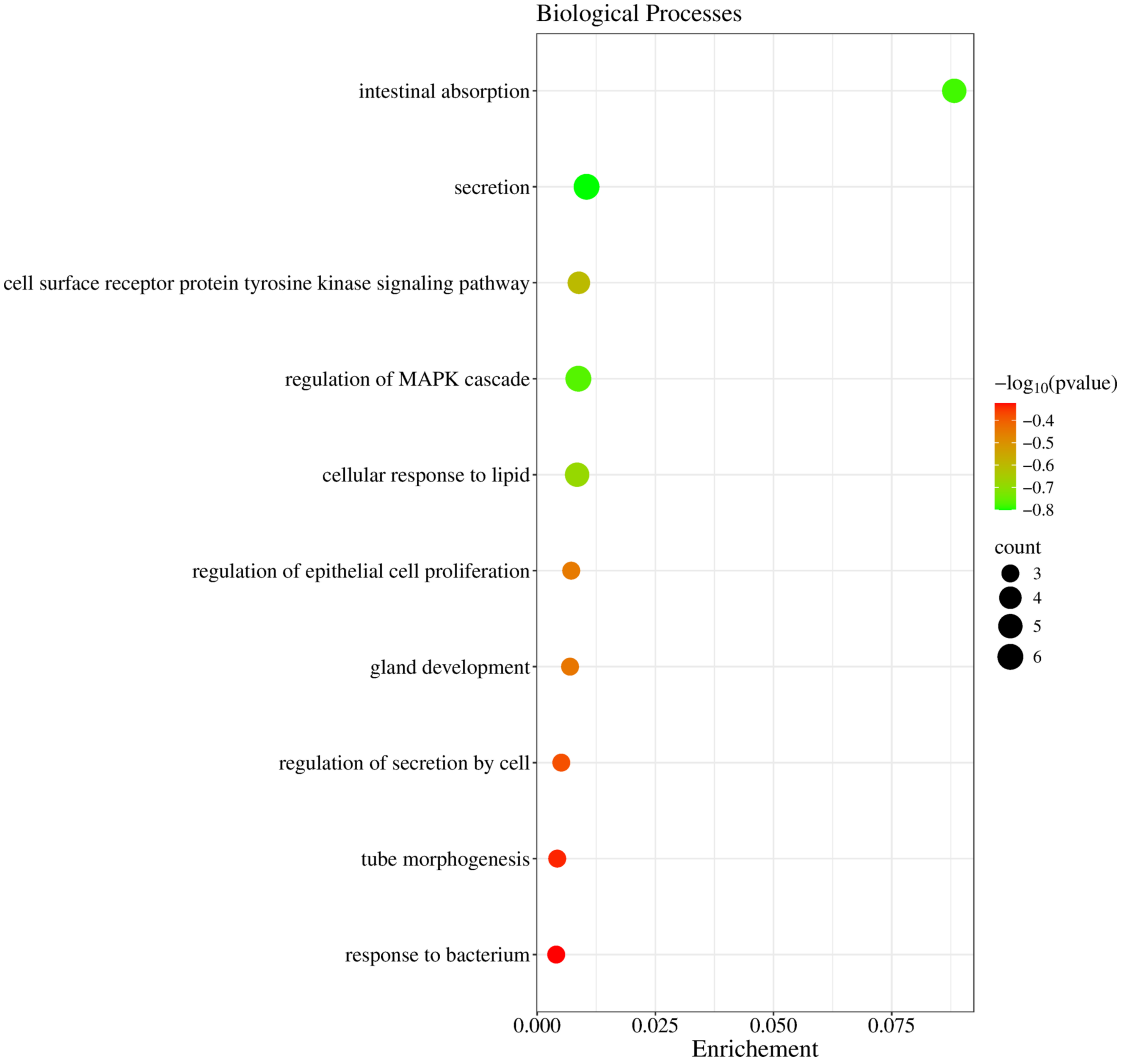
Bioinformatic plot of the top 10 biological processes involved in gut dysbiosis.

Figure 6 presents the biological processes involved in gut dysbiosis and targeted by the compounds. The biological processes obtained from the analysis mainly refer to several signal transduction pathways, the significance and enrichment levels of which vary. Secretory (log10(p, value) = 0.4, Enrichment = 0.075) is the most highly enriched process, followed by cell surface receptor protein tyrosine kinase signaling pathway (log10(p, value) = 0.6, Enrichment = 0.065) and regulation of MAPK cascade (log10(p, value) = 0.7, Enrichment = 0.06), which are also very highly enriched. However, these values are not statistically significant according to the standard threshold (generally *p* < 0.05, i.e., −log₁₀(p) > 1.3).

#### 3.3.5 Compound target biological processes

**Figure 7:**
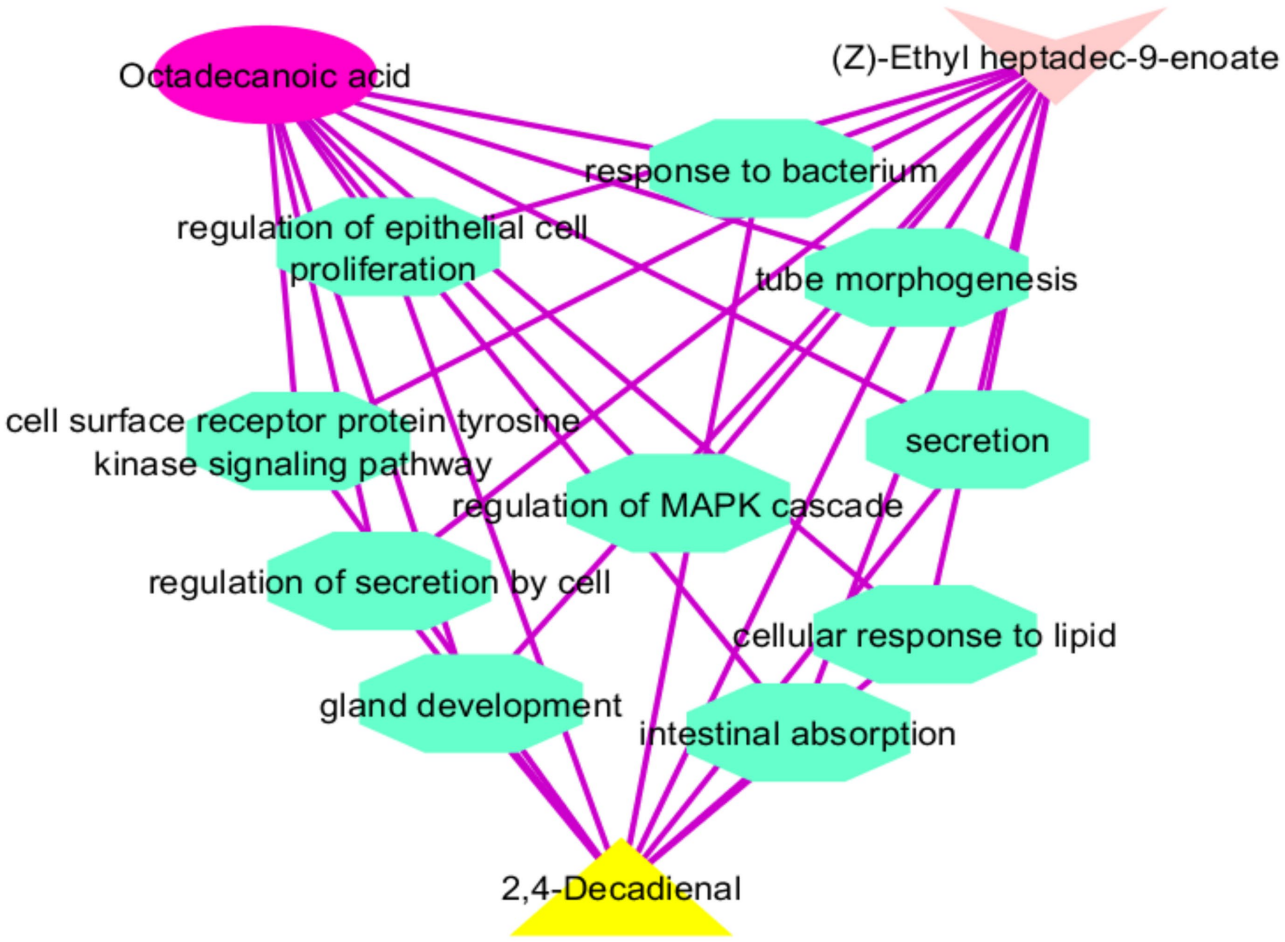
Compounds target biological processes.

The compound-target biological process network highlights: regulation of secretion by cell (rank 1), regulation of epithelial cell proliferation (rank 2), response to bacterium (rank 3), gland development (rank 4), tube morphogenesis (rank 5), cellular response to lipid (rank 6), cell surface receptor protein tyrosine kinase signaling pathway (rank 7), intestinal absorption (rank 8), and secretion (rank 9), as the most central biological processes. These processes have the same topological properties (degree = 3.0, betweenness = 0.8667, closeness = 0.5714), which means they have key roles in the network and are very interconnected. On the other hand, regulation of the MAPK cascade (rank 10) has slightly lower connectivity (degree = 2.0, betweenness = 0.2, closeness = 0.5217), and this may be indicative of its role as a regulatory mechanism that supports and is still quite relevant in mediating the biological effects induced by compounds (Table 6; Figure 7).

**Table 6:** Top 10 biological processes targeted by the compounds.

Rank	Biological processes	Degree	Betweenness	Closeness
1	Regulation of secretion by the cell	Degree: 3.0	Betweenness: 0.8666667	Closeness: 0.5714286
2	Regulation of epithelial cell proliferation	Degree: 3.0	Betweenness: 0.8666667	Closeness: 0.5714286
3	Response to the bacterium	Degree: 3.0	Betweenness: 0.8666667	Closeness: 0.5714286
4	Gland development	Degree: 3.0	Betweenness: 0.8666667	Closeness: 0.5714286
5	Tube morphogenesis	Degree: 3.0	Betweenness: 0.8666667	Closeness: 0.5714286
6	Cellular response to lipid	Degree: 3.0	Betweenness: 0.8666667	Closeness: 0.5714286
7	Cell surface receptor protein tyrosine kinase signaling pathway	Degree: 3.0	Betweenness: 0.8666667	Closeness: 0.5714286
8	Intestinal absorption	Degree: 3.0	Betweenness: 0.8666667	Closeness: 0.5714286
9	Secretion	Degree: 3.0	Betweenness: 0.8666667	Closeness: 0.5714286
10	Regulation of MAPK cascade	Degree: 2.0	Betweenness: 0.2	Closeness: 0.5217391

#### 3.3.6 Transcription factor and kinases targeted by the compounds

**Figure 8:**
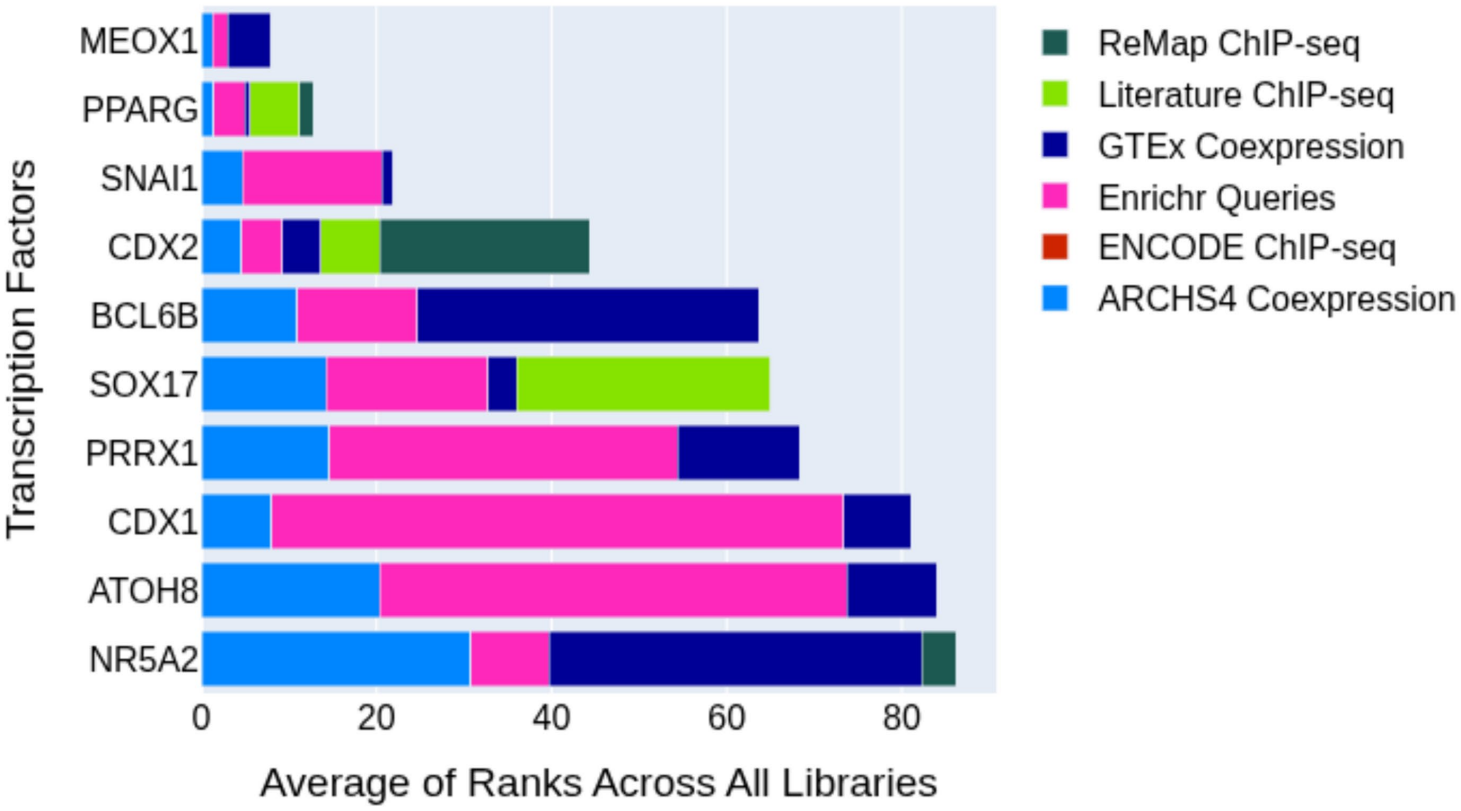
Bioinformatic plot of the top 10 transcription factors involved in gut dysbiosis.

Figure 8 and Table 7 show the top 10 Transcription Factors involved in Gut dysbiosis. The transcription factors topping the list in the network analysis are:, MEOX1 with an average rank of 8 and overlapping genes such as FABP4, SCD, MMP2, KDR, and PTGS2;, PPARG with an average rank of 12.8 and overlapping genes like FABP4, SCD, VDR, MMP2, KDR, FFAR4, GPBAR1, and PTGS2;, SNAI1 (average rank 22) overlapping FABP4, VDR, MMP2, KDR, GPBAR1, and PTGS2;, CDX2 (average rank 44.4) overlapping FABP2, VDR, MMP2, FFAR4, and KDR;, BCL6B (average rank 63.67) overlapping MMP2, KDR, and PTGS2;, SOX17 (average rank 65) overlapping FABP4, MMP2, KDR, and PTGS2;, PRRX1 (average rank 68.33) overlapping MMP2 and PTGS2;, CDX1 (average rank 81) overlapping FABP2, VDR, and PTGS2;, ATOH8 (average rank 84) overlapping MMP2, KDR, PTGS2, and FKBP5; and, NR5A2 (average rank 86.25) overlapping FABP2, GPR119, VDR, RORC, KDR, and PTGS2.

**Table 7:** Top 10 transcription factors involved in gut dysbiosis.

Rank	Transcription factor	Avg Rank	Library ranks	Overlapping genes
1	MEOX1	8	ARCHS4 Coexpression: 4, Enrichr Queries: 5, GTEx Coexpression: 15	FABP4, SCD, MMP2, KDR, PTGS2
2	PPARG	12.8	Literature ChIP-seq: 28, ARCHS4 Coexpression: 7, Enrichr Queries: 18, ReMap ChIP-seq: 8, GTEx Coexpression: 3	FABP4, SCD, VDR, MMP2, KDR, FFAR4, GPBAR1, PTGS2
3	SNAI1	22	ARCHS4 Coexpression: 14, Enrichr Queries: 48, GTEx Coexpression: 4	FABP4, VDR, MMP2, KDR, GPBAR1, PTGS2
4	CDX2	44.4	Literature ChIP-seq: 34, ARCHS4 Coexpression: 23, Enrichr Queries: 23, ReMap ChIP-seq: 120, GTEx Coexpression: 22	FABP2, VDR, MMP2, FFAR4, KDR
5	BCL6B	63.6667	ARCHS4 Coexpression: 33, Enrichr Queries: 41, GTEx Coexpression: 117	MMP2, KDR, PTGS2
6	SOX17	65	Literature ChIP-seq: 116, ARCHS4 Coexpression: 57, Enrichr Queries: 74, GTEx Coexpression: 13	FABP4, MMP2, KDR, PTGS2
7	PRRX1	68.3333	ARCHS4 Coexpression: 44, Enrichr Queries: 119, GTEx Coexpression: 42	MMP2, PTGS2
8	CDX1	81	ARCHS4 Coexpression: 24, Enrichr Queries: 196, GTEx Coexpression: 23	FABP2, VDR, PTGS2
9	ATOH8	84	ARCHS4 Coexpression: 61, Enrichr Queries: 160, GTEx Coexpression: 31	MMP2, KDR, PTGS2, FKBP5
10	NR5A2	86.25	ARCHS4 Coexpression: 123, Enrichr Queries: 36, ReMap ChIP-seq: 16, GTEx Coexpression: 170	FABP2, GPR119, VDR, RORC, KDR, PTGS2

#### 3.3.7 Kinases targeted by the compounds

**Figure 9:**
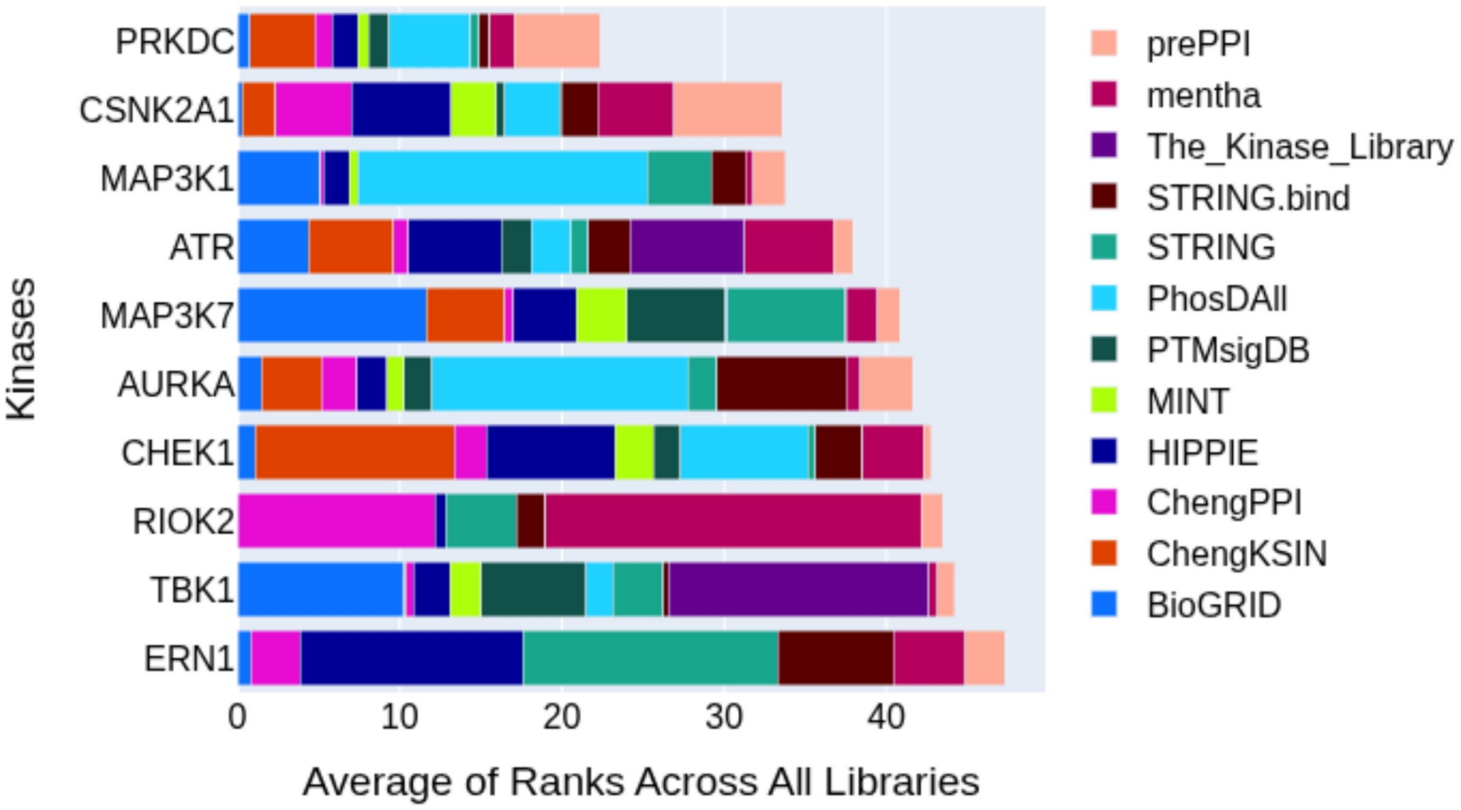
Bioinformatic plot of top 10 kinase involved in gut dysbiosis.

Figure 9 and Table 8 show the ranked kinases targeted by the compound and involved in Gut dysbiosis. Protein kinase enrichment analysis indicated PRKDC as the highest-ranked kinase with an average rank of 22.36, which was linked to the common genes such as APP, GSK3B, MCM7, KIF11, PHB2, RPL7A, RUVBL2, RUVBL1, EP300, and GTF2I. CSNK2A1 came next with an average rank of 33.55, having common genes such as APP, GSK3B, MCM7, KIF11, PHB2, RPL9, RPS4X, RPL7A, RUVBL2, and RUVBL1. MAP3K1 came third (average score 33.78) with overlapping genes GSK3B, APP, HDAC3, SF3B3, HDAC1, DHX9, SRSF1, KIF11, RPS4X, and BAG2. ATR had an average rank of 37.91, sharing with APP, GSK3B, HDAC3, MCM7, HDAC1, DHX9, SRSF1, KIF11, PHB2, and RPL9. MAP3K7 was the fifth, ranked with an average rank of 40.82, overlapping with APP, GSK3B, HDAC3, MCM7, HDAC1, DHX9, SRSF1, PHB2, RPL7A, and RUVBL2. AURKA (average rank 41.64), CHEK1 (42.73), RIOK2 (43.5), TBK1 (44.17), and ERN1 (47.29) came next, with each kinase sharing a list of genes with the others, such as APP, GSK3B, HDAC3, MCM7, HDAC1, DHX9, SRSF1, KIF11, PHB2, RPL9, etc. The kinase rankings were generated on the basis of a range of databases, including STRING, ChengPPI, PhosDAll, BioGRID, HIPPIE, ChengKSIN, MINT, mentha, prePPI, and PTMsigDB.

**Table 8:** Top 10 of ranked kinase involved in Gut dysbiosis.

Rank	Kinase	Avg Rank	Library ranks	Overlapping genes
1	PRKDC	22.3636	STRING.bind: 7, ChengPPI: 11, PhosDAll: 55, BioGRID: 8, HIPPIE: 18, ChengKSIN: 45, STRING: 6, MINT: 7, mentha: 17, prePPI: 58,	APP, GSK3B, MCM7, KIF11, PHB2, RPL7A, RUVBL2, RUVBL1, EP300, GTF2I
2	CSNK2A1	33.5455	STRING.bind: 24, ChengPPI: 51, PhosDAll: 37, BioGRID: 3, HIPPIE: 68, ChengKSIN: 23, STRING: 2, MINT: 30, mentha: 52, prePPI: 73,	APP, GSK3B, MCM7, KIF11, PHB2, RPL9, RPS4X, RPL7A, RUVBL2, RUVBL1
3	MAP3K1	33.7778	STRING.bind: 19, ChengPPI: 2, PhosDAll: 160, BioGRID: 46, HIPPIE: 14, STRING: 36, MINT: 5, mentha: 4, prePPI: 18	GSK3B, APP, HDAC3, SF3B3, HDAC1, DHX9, SRSF1, KIF11, RPS4X, BAG2
4	ATR	37.9091	STRING.bind: 28, ChengPPI: 10, PhosDAll: 27, BioGRID: 48, HIPPIE: 63, ChengKSIN: 58, STRING: 12, The_Kinase_Library: 78,	APP, GSK3B, HDAC3, MCM7, HDAC1, DHX9, SRSF1, KIF11, PHB2, RPL9
5	MAP3K7	40.8182	STRING.bind: 1, ChengPPI: 6, PhosDAll: 1, BioGRID: 128, HIPPIE: 43, ChengKSIN: 53, STRING: 80, MINT: 34, mentha: 21, prePPI: 15,	APP, GSK3B, HDAC3, MCM7, HDAC1, DHX9, SRSF1, PHB2, RPL7A, RUVBL2
6	AURKA	41.6364	STRING.bind: 88, ChengPPI: 24, PhosDAll: 173, BioGRID: 16, HIPPIE: 20, ChengKSIN: 41, STRING: 20, MINT: 12, mentha: 9, prePPI: 36, PTMsigDB: 19	GSK3B, APP, HDAC3, MCM7, HDAC1, DHX9, SRSF1, KIF11, RPS4X, RUVBL2
7	CHEK1	42.7273	STRING.bind: 32, ChengPPI: 22, PhosDAll: 87, BioGRID: 12, HIPPIE: 87, ChengKSIN: 135, STRING: 5, MINT: 26, mentha: 41, prePPI: 5,	APP, GSK3B, HDAC3, SF3B3, MCM7, HDAC1, DHX9, SRSF1, KIF11, PHB2
8	RIOK2	43.5	STRING.bind: 11, ChengPPI: 73, HIPPIE: 4, STRING: 26, mentha: 139, prePPI: 8	APP, GSK3B, MCM7, HDAC1, DHX9, SRSF1, RPL9, RPS4X,
9	TBK1	44.1667	STRING.bind: 4, ChengPPI: 5, PhosDAll: 20, BioGRID: 123, HIPPIE: 27, ChengKSIN: 2, STRING: 37, MINT: 23, The_Kinase_Library: 192, mentha: 7, prePPI: 12, PTMsigDB: 78	APP, GSK3B, HDAC3, MCM7, HDAC1, DHX9, SRSF1, KIF11, RUVBL2, RUVBL1
10	ERN1	47.2857	STRING.bind: 50, ChengPPI: 21, BioGRID: 6, HIPPIE: 96, STRING: 110, mentha: 31, prePPI: 17	HSPA9, APP, GSK3B, HSPA8, MAP2K1, HSP90AA1, CREBBP, CSNK2A1, HSPA5,

#### 3.3.8 Protein–protein interaction network integrates transcription factors and kinases

**Figure 10:**
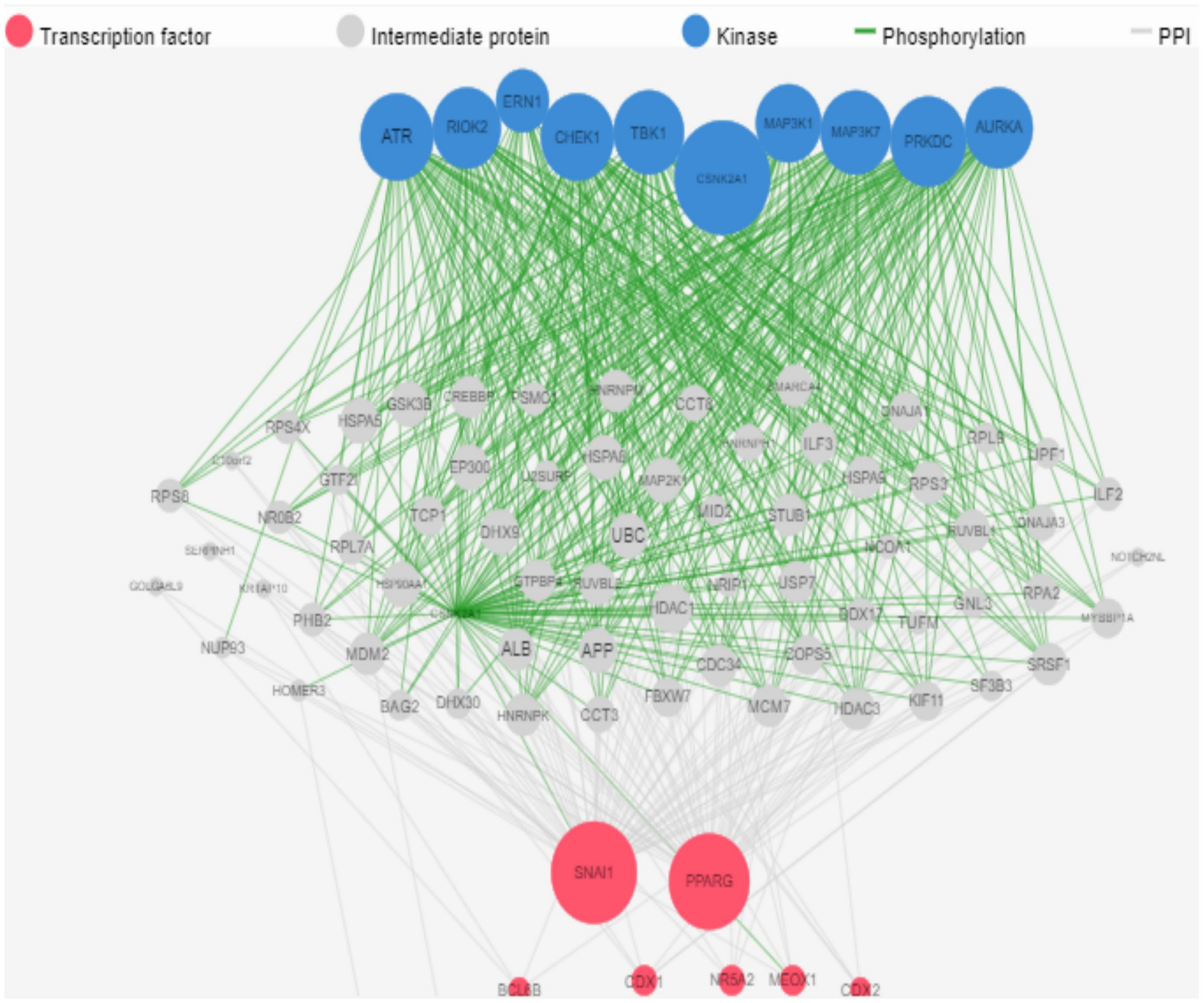
Protein–protein interaction network integrates transcription factors and kinases.

Figure 10 shows that aside from transcription factors (red nodes) and kinases (blue nodes), the protein–protein interaction (PPI) network also includes intermediate proteins (gray nodes). All these nodes illustrate their regulatory and interaction relationships. Kinases, such as CSNK2A1, MAP3K1, MAP3K7, PRKDC, AURKA, ATR, CHEK1, TBK1, RIOK2, and ERN1, make up a tightly interconnected cluster in the upper part of the network, thus highlighting their major role in phosphorylation events (green edges). Further down, the transcription factors SNAI1 and PPARG appear as bigger red nodes, which means they have many connections and are likely to have a strong regulatory influence on their downstream targets. Besides them, other transcription factors such as CDX1, NR5A2, MEOX1, and CDX2 are also present but with smaller node sizes, reflecting their relatively lower network rank. The gray intermediate proteins serve as connectors that facilitate protein–protein interactions (light gray edges) between kinases and transcription factors, thus suggesting a complex regulatory cascade.

### 3.4 Protein structures validation

**Figure 11:**
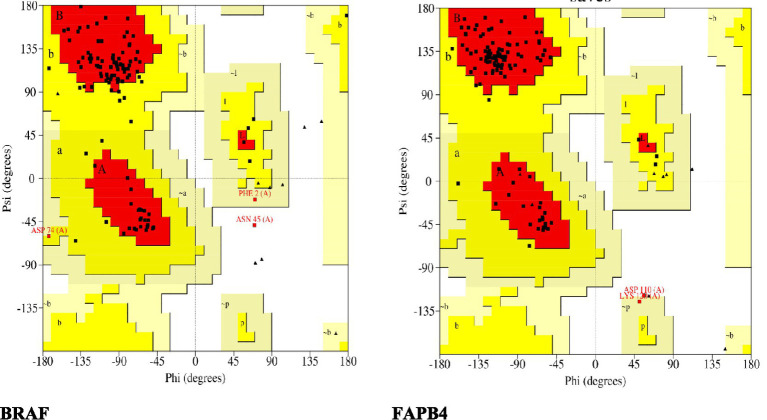
Ramachandran plot of target proteins.

Figure 11 shows the Ramachandran plot of 3D structures of target proteins. Concerning FABP4, a very high percentage of residues (92.4%) are situated in the most favored regions, which mainly correspond to helical and sheet conformations, and this is above the generally accepted quality threshold of 90% and hence signifies a nicely refined structure. Another 5.9% of the residues lie in the additionally allowed regions, and only a very minor proportion (0.8%) belongs to the generously allowed regions. Interestingly, one residue (0.8%) is located in the disallowed region, which may require further scrutiny to understand whether it is a sign of local structural strain or functional relevance, or simply a modeling error. Regarding the BRAF protein, 91.6% of the 191 residues that exclude glycines and prolines are located in the most favored regions, while the remaining 8.4% are in the additionally allowed regions. All residues play conformations that fit within the space allowed by the Ramachandran plot, with none of them residing either in the generously allowed or disallowed regions. The well-defined clustering of amino acid residues in the canonical helix and sheet regions is a straightforward sign of the arrangements of the tertiary structure. Glycine and proline residues enable the conformational flexibility corresponding to their locations through separate clustering.

### 3.5 Molecular docking results

Molecular docking studies of FABP4 with three fatty acid derivatives showed different binding interactions for each of the compounds. 2,4-Decadienal gave a binding energy of −5.1 kcal/mol. It mainly interacted via hydrophobic contacts with residues Phe16, Ala17, Met20, Val25, and also formed hydrogen bonds with Thr79 and Arg126. The ligand was thus stabilized within the binding site through a combination of van der Waals and polar interactions. (Z)-Ethyl heptadec-9-enoate produced a binding energy of −5.8 kcal/mol. The compound binds to residues Phe16, Ala33, Phe57, Ala75, Met20, Val115, and forms hydrogen bonds particularly with Thr79 and Arg126. The ligand was mostly stabilized through hydrophobic contacts supported by hydrogen bonds. Octadecanoic acid, through an energy of −5.8 kcal/mol, has a similar binding mode to (Z)- ethyl heptadec-9-enoate, involving residues Phe16, Ala33, Phe57, Ala75, Met20, and Val115, and forming hydrogen bonds with Thr79 and Arg126. The ligand stabilization in this case is mainly through hydrophobic and polar interactions. The standard drug Bms-309403 had the strongest binding energy at −10.4 kcal/mol. It established a number of hydrogen bonds with Arg128, Tyr128, Met125, and Ala76, and also hydrophobic interactions with the surrounding residues in agreement with the high affinity (Table 9).

**Table 9:** 2D and 3D interactions between the ligands and FABP4 protein.

Protein	FABP4			
Compounds	2,4-Decadienal	(Z)-Ethyl heptadec-9-enoate	Octadecanoic acid	Bms-309403
3D	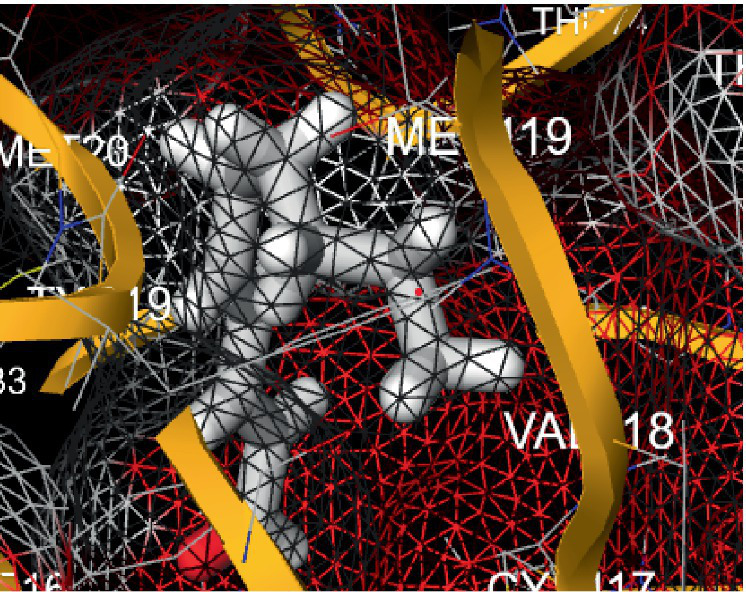	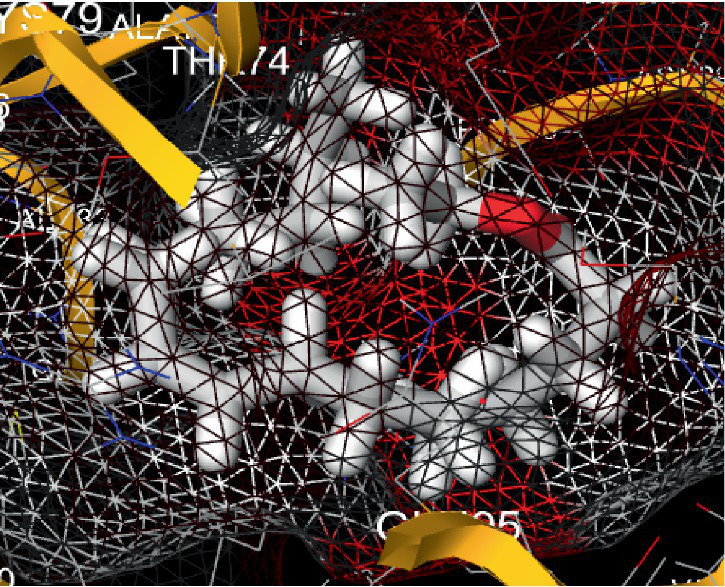	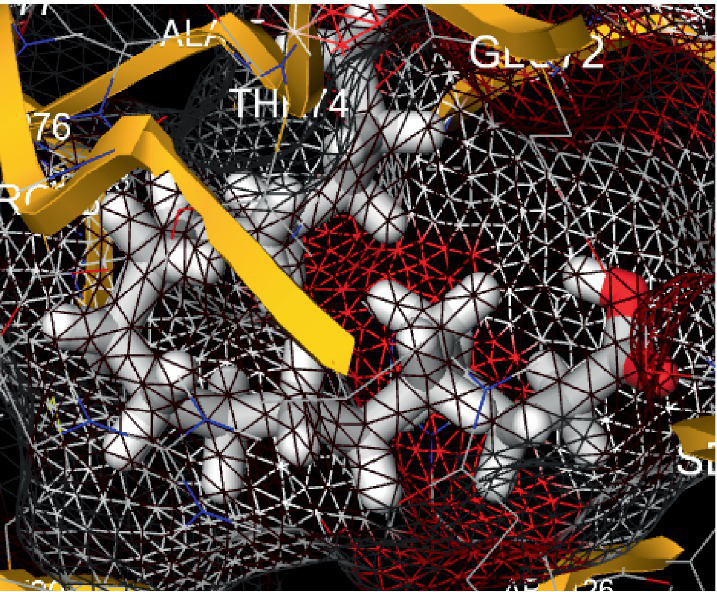	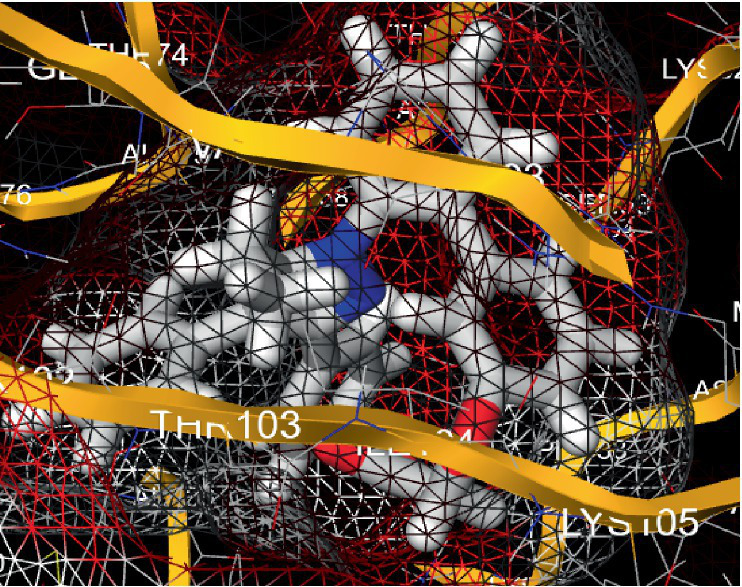
	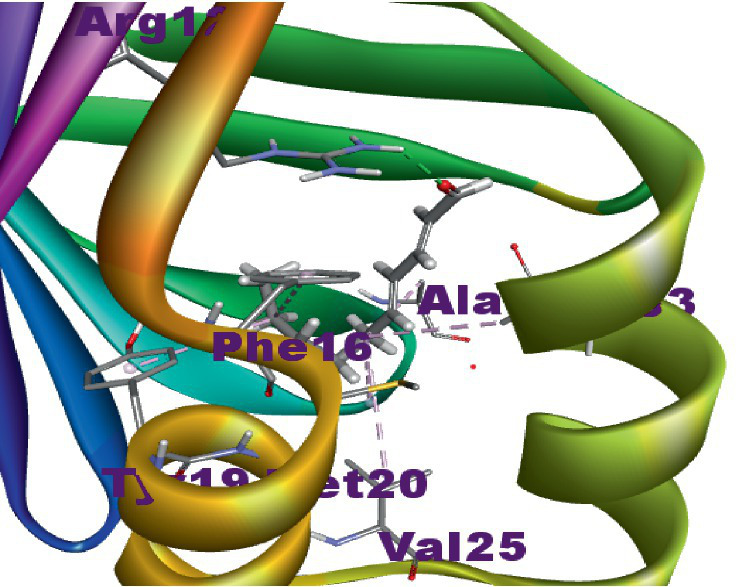	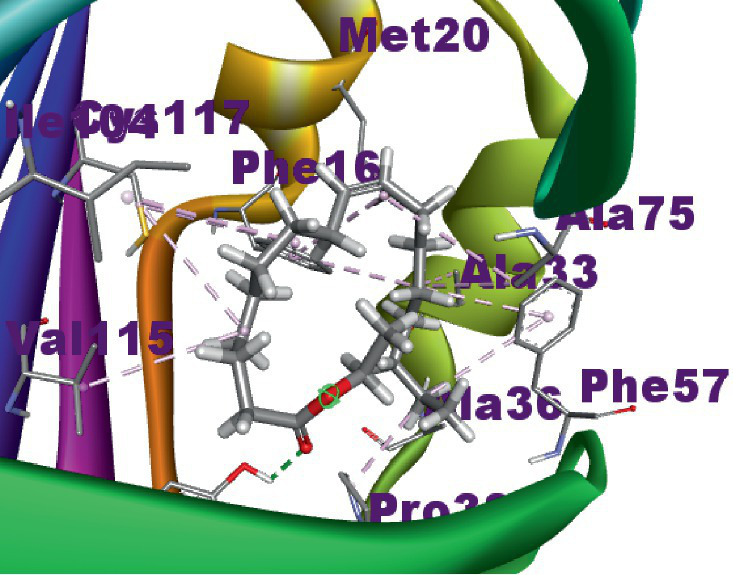	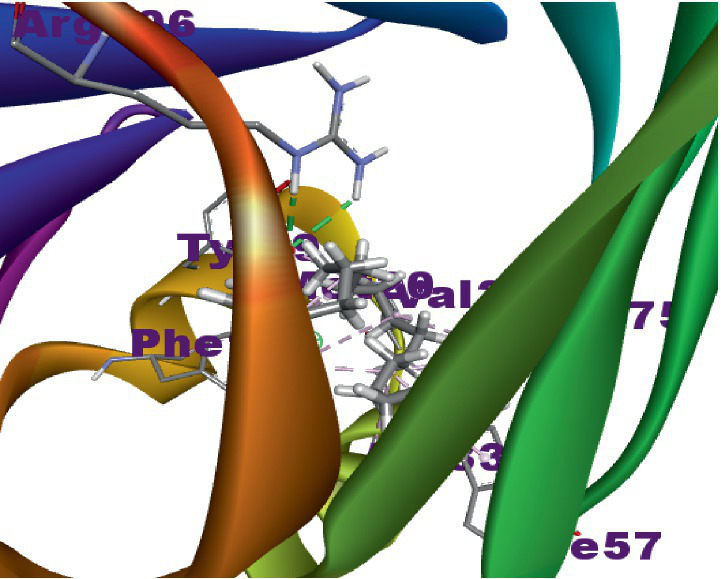	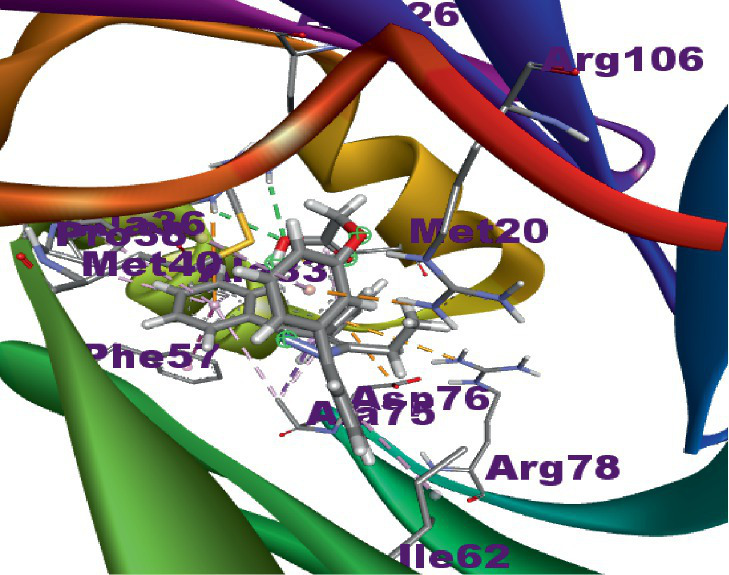
2D	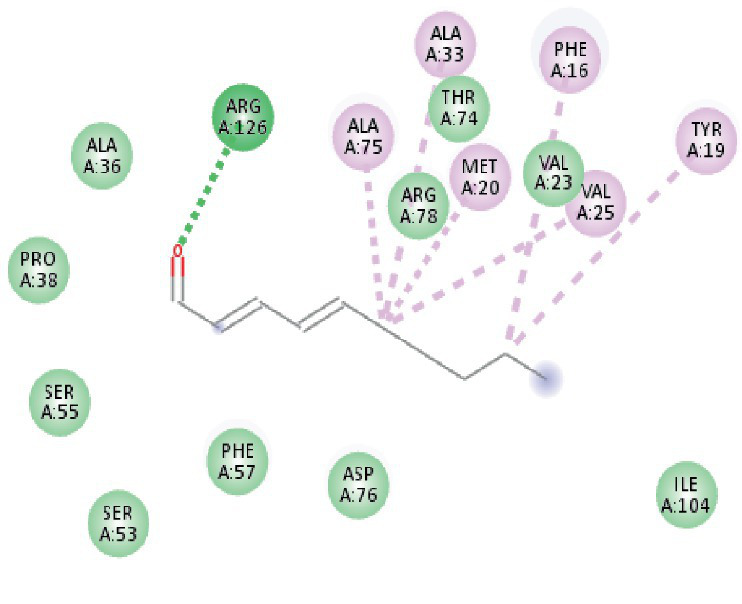	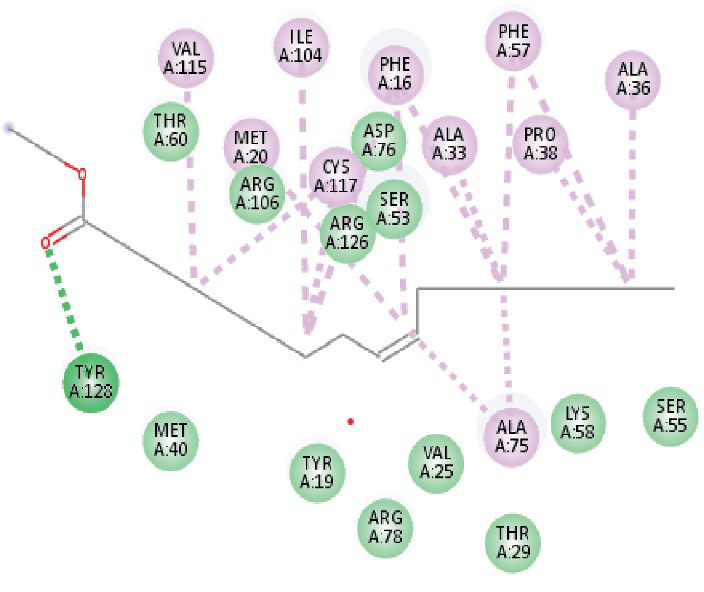	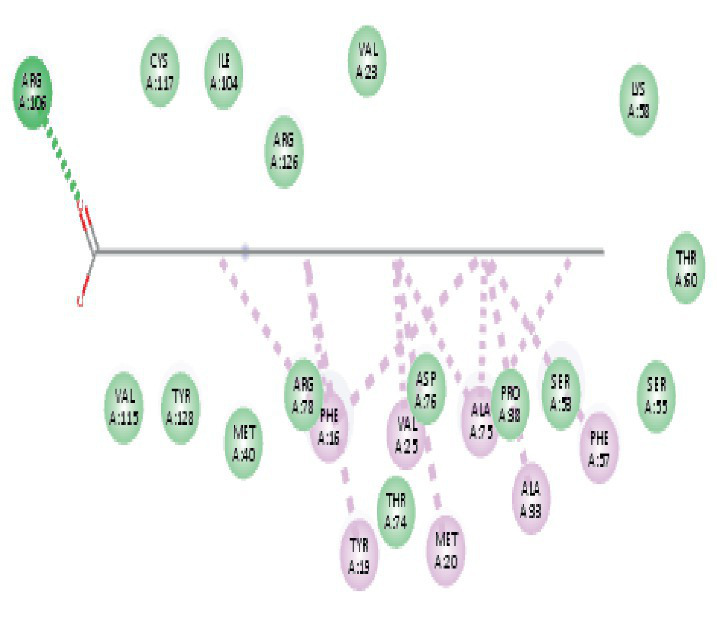	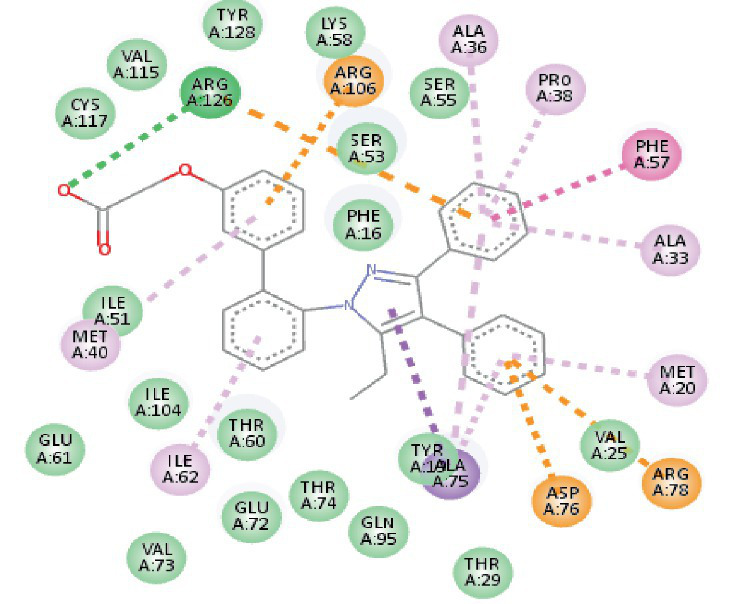
Energies	−5.1	−5.8	−5.8	−10.4

The molecular docking of 2,4-decadienal-(Z)-ethyl heptadec, 9, enoate, and octadecanoic acid targeting the BRAF protein showed good binding to the active site, which was similar to the binding of the reference inhibitor vemurafenib. 2,4-decadienal had a docking score of −5.5 kcal/mol and was mostly maintained by hydrophobic and alkyl interactions with crucial amino acid residues like Trp84, Phe, Val, Leu, and Ile that are helping the ligand to be stabilized inside the pocket. The molecule (Z)-ethyl heptadec-9-enoate had the highest interaction energy of −6.2 kcal/mol which indicates that it could be fit for BRAF very closely; the long hydrocarbon chain of the molecule facilitates extensive hydrophobic (alkyl and alkyl) interactions with the residues such as Phe, Val, Ala, Ile, and Leu, and also the close contacts with Trp84, which contributes to the stable binding. Octadecanoic acid had a binding energy of −5.8 kcal/mol and was stabilized through hydrophobic contacts with residues like Leu, Val, Ile, and Phe, and a hydrogen bond through the carboxyl group with a polar or charged residue such as Lys or Asp that further stabilizes the complex. The reference drug vemurafenib showed binding energy of −5.9 kcal/mol and was very well bound to critical active-site residues, including Trp84, Phe, Leu, Val, and Lys, through a combination of hydrogen bonds, stacking, and alkyl (Table 10). Rifaximin, as a treatment for intestinal dysbiosis, has not shown an affinity for target proteins with positive binding energies (Table 11).

**Table 10:** 2D and 3D interactions between the compounds and BRAF protein.

Protein	BRAF.			
Compounds	2,4-Decadienal	(Z)-Ethyl heptadec-9-enoate	Octadecanoic acid	Vemurafenib
3D	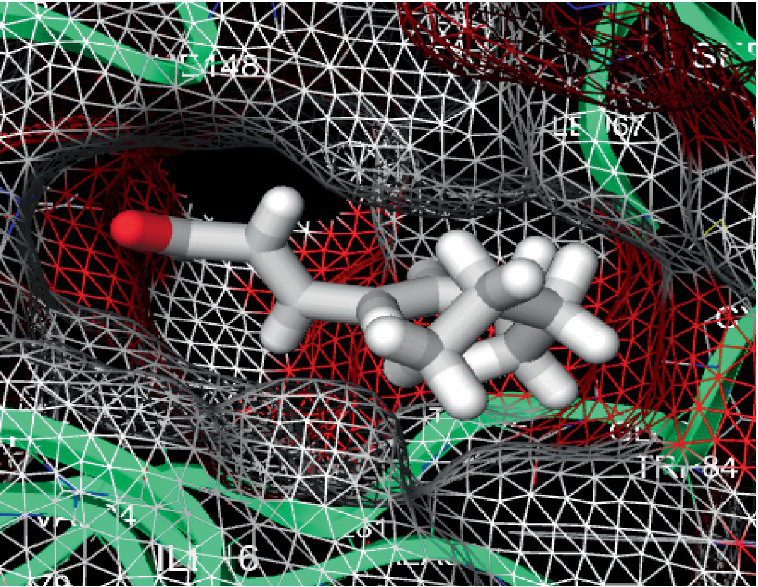	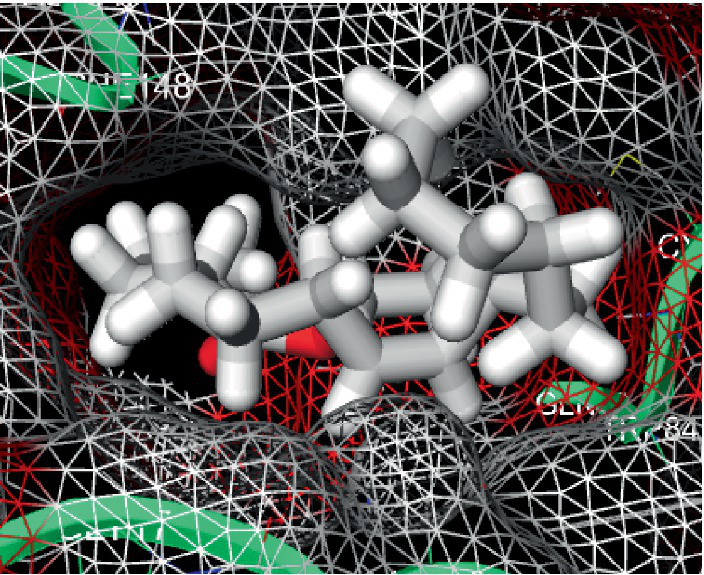	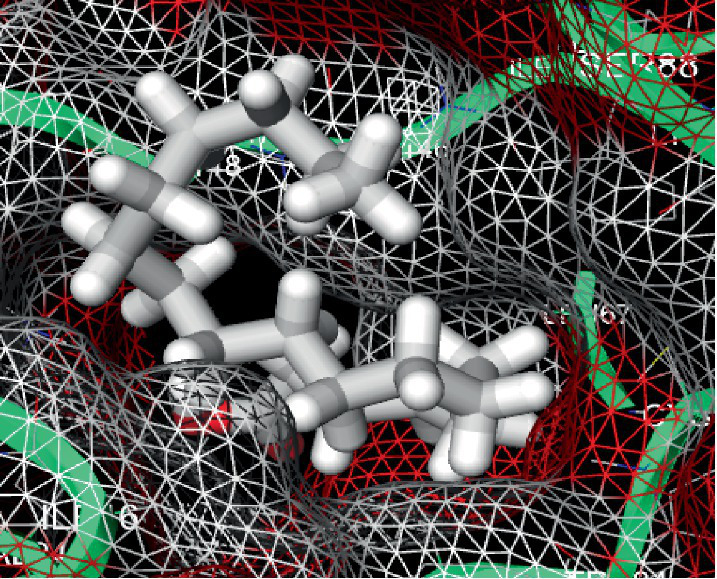	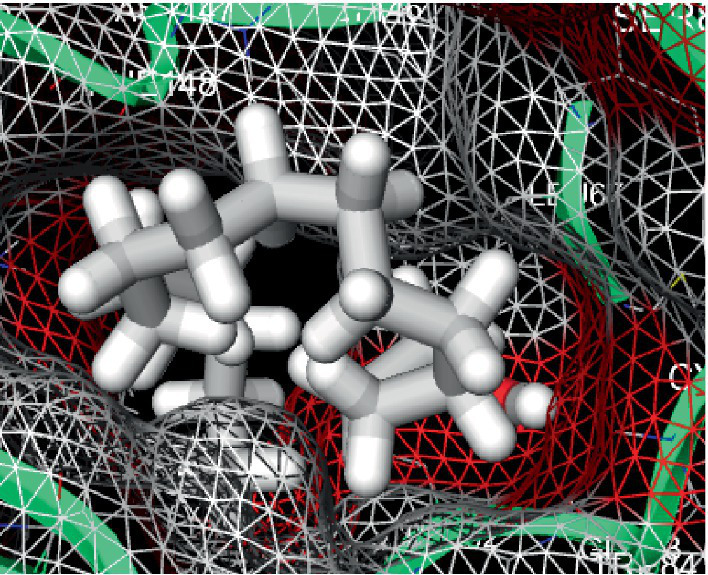
	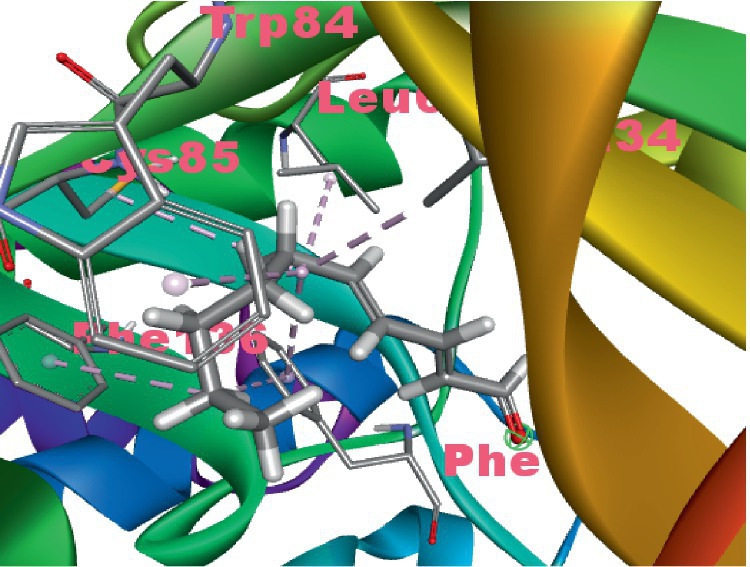	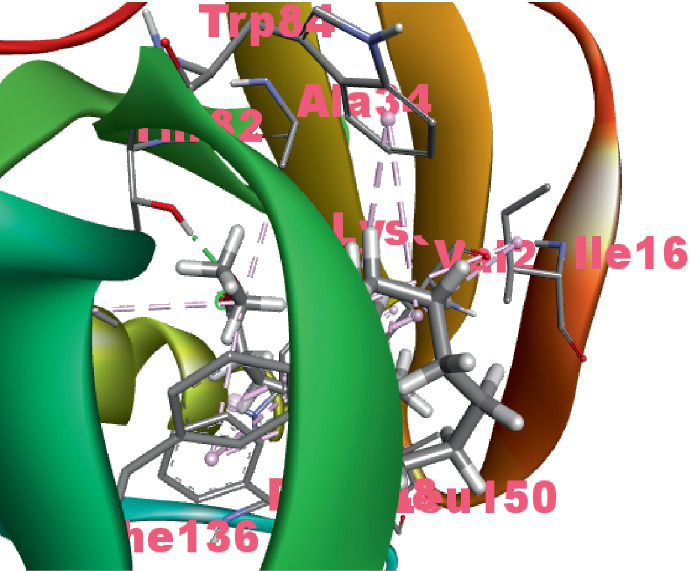	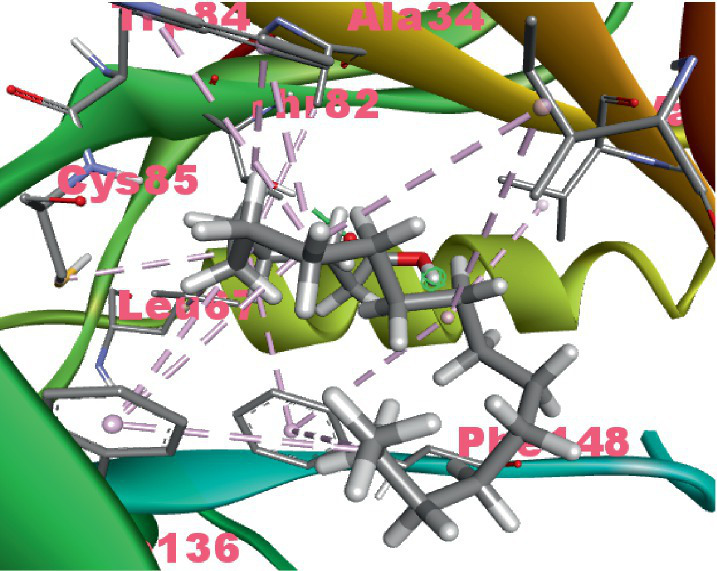	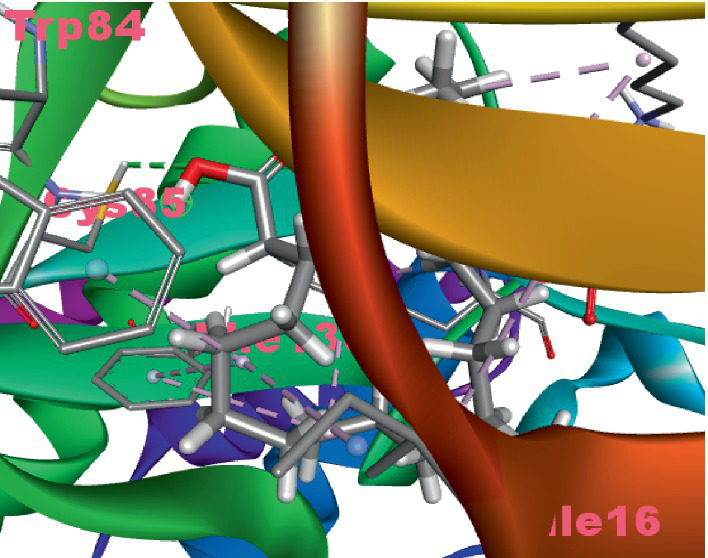
2D	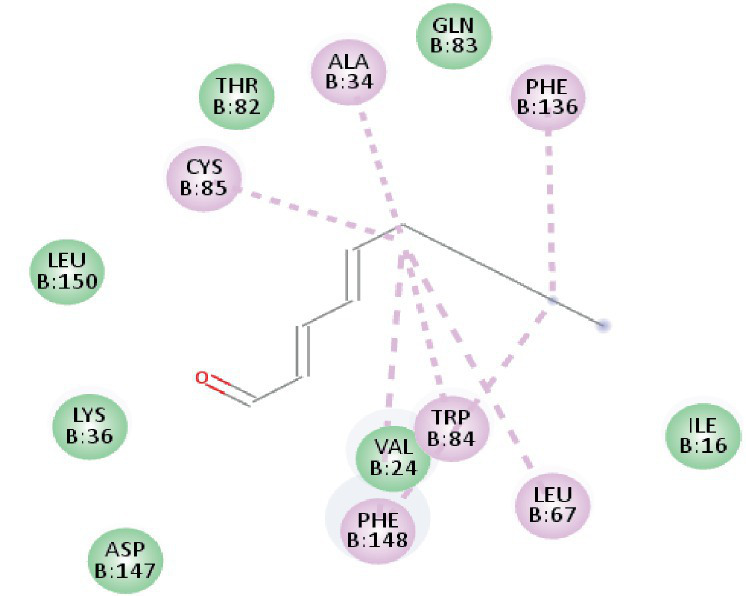	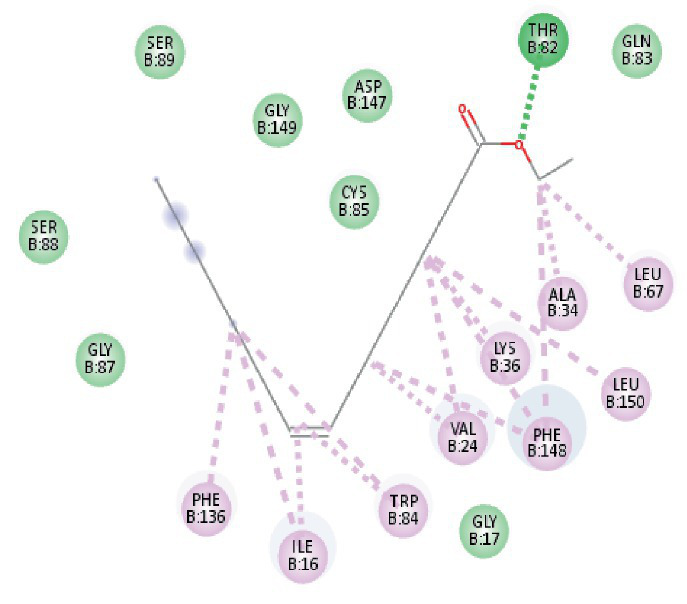	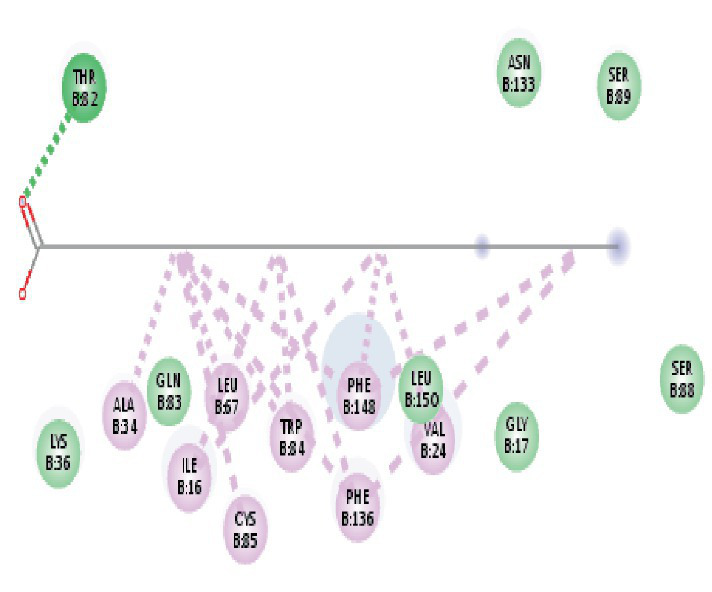	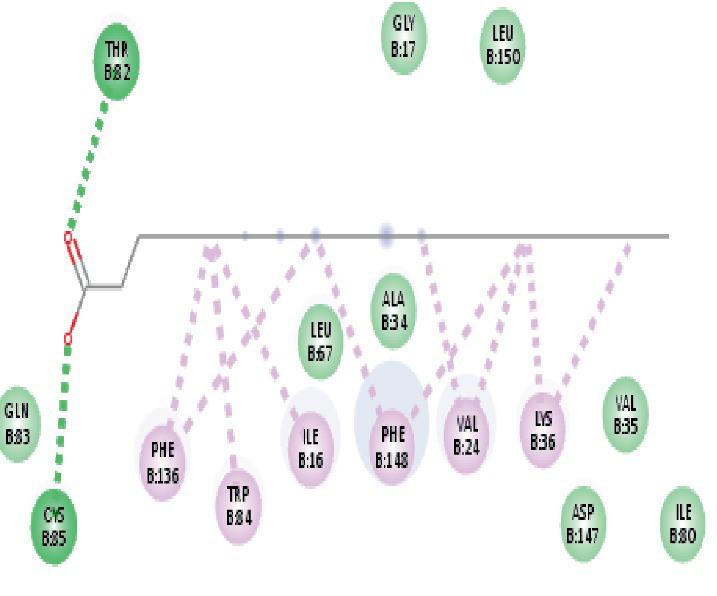
Energies	−5.5	−6.2	−5.8	−5.9

**Table 11:** 2D and 3D interactions between Rifaximin and BRAF, and FABP4 proteins.

	3D		2D	Energy
BRAF	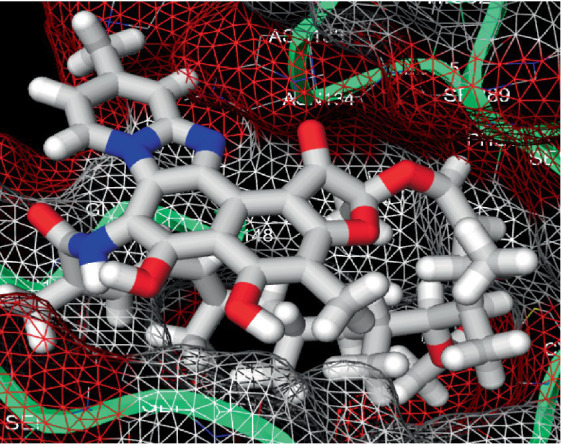	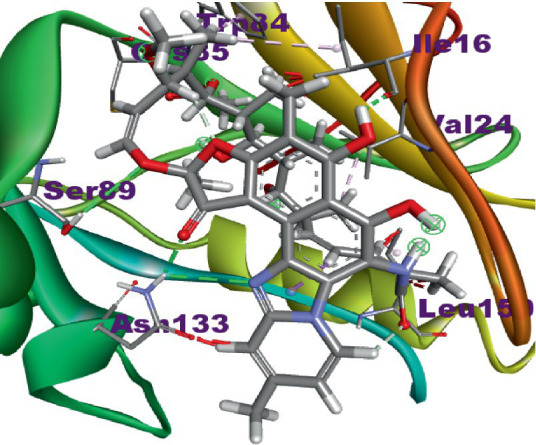	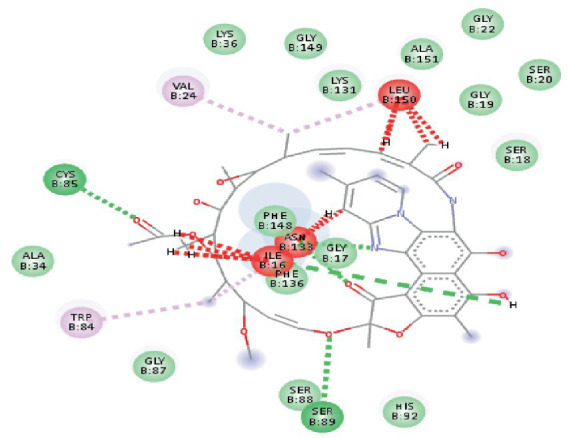	10.8
FABP4	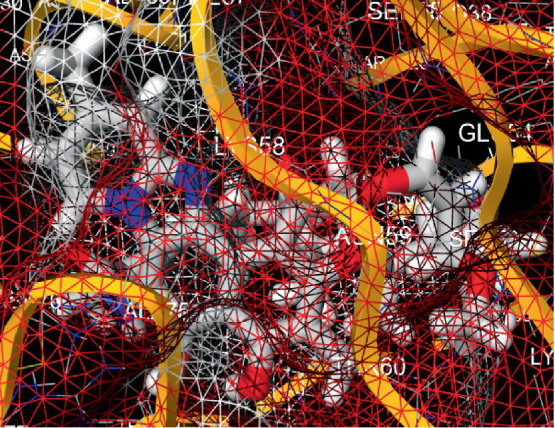	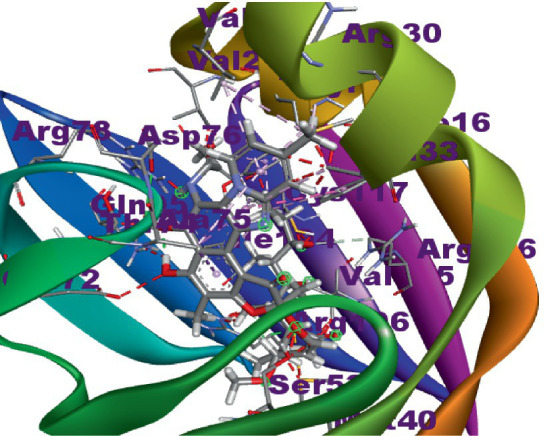	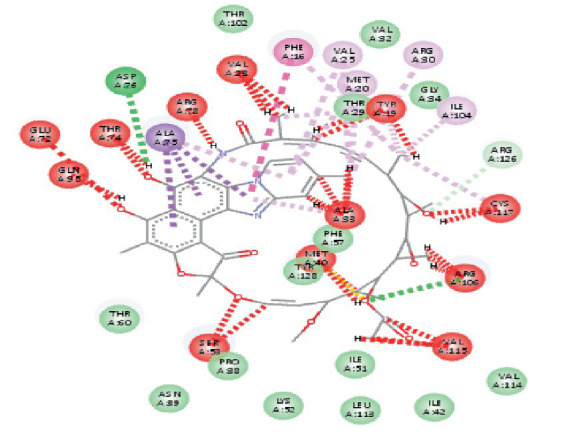	40.1
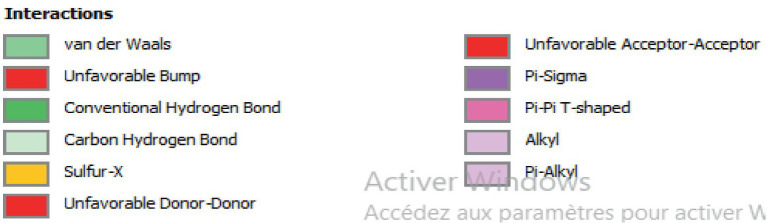				

### 3.6 Normal mode analysis data

**Figure 12:**
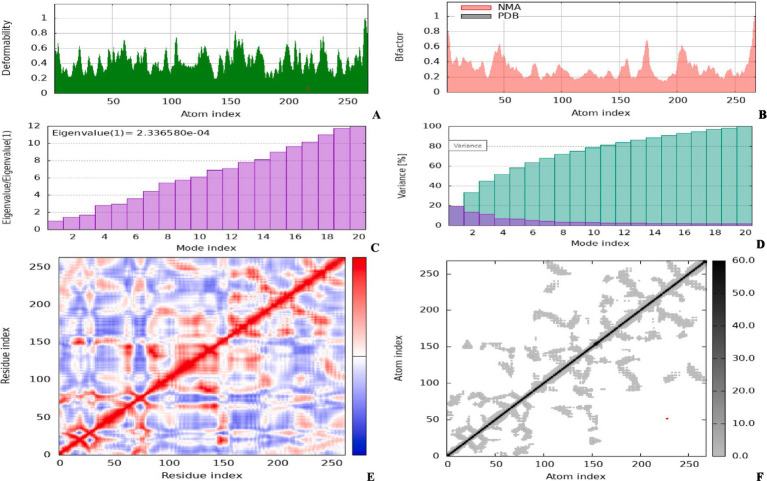
Normal mode analysis of best-docked complexes. **(A)** Deformability profile, **(B)** B factor plot, **(C)** Eigenvalue plot, **(D)** Variance plot, **(E)** Correlation matrix, **(F)** Elastic network analysis.

Figure 12 shows the Normal mode analysis result of the best-docked complexes. According to this Figure, the NMA analyzed (Z)-ethyl heptadec-9-enoate-BRAF complex reveals a globally stable and well-organized system on the molecular level. The deformability profile (Figure 12A) mainly displays low to moderate flexibility throughout the protein chain, with only a few peaks referring to loop or solvent-exposed regions, which implies that the binding of the ligand does not cause a major destabilization of the protein structure. This fact is supported by the B-factor plot (Figure 12B) as well, where the values obtained from Normal Mode Analysis agree with those of the PDB structure, thus pointing to a somewhat stable protein core, especially in the vicinity of the binding site. The eigenvalue plot (Figure 12C) shows a very low first eigenvalue (2.34 10, pre-factor excluded), an indication that the collective motions of the protein require very little energy and that thus the complex can undergo such local adjustments as are essential for the function without destabilizing the structure. Furthermore, the variance plot (Figure 12D) bears evidence that the main total variance is a result of the first few low-frequency modes, which is typical of a biological complex stabilized by controlled global motions. The residue cross-correlation matrix (Figure 12E) displays strong positive correlations along the diagonal and several distinct regions of long-range correlated and anti-correlated motions, which together indicate a dynamic protein whose distant parts can communicate efficiently and whose internal motions become concerted upon binding of the ligand. Finally, the rigidity and elastic network analysis (Figure 12F) highlights well-defined rigid clusters, particularly surrounding the active site, indicating that the ligand contributes to reinforcing local structural stability.

### 3.7 Pharmacophore analysis results

**Figure 13:**
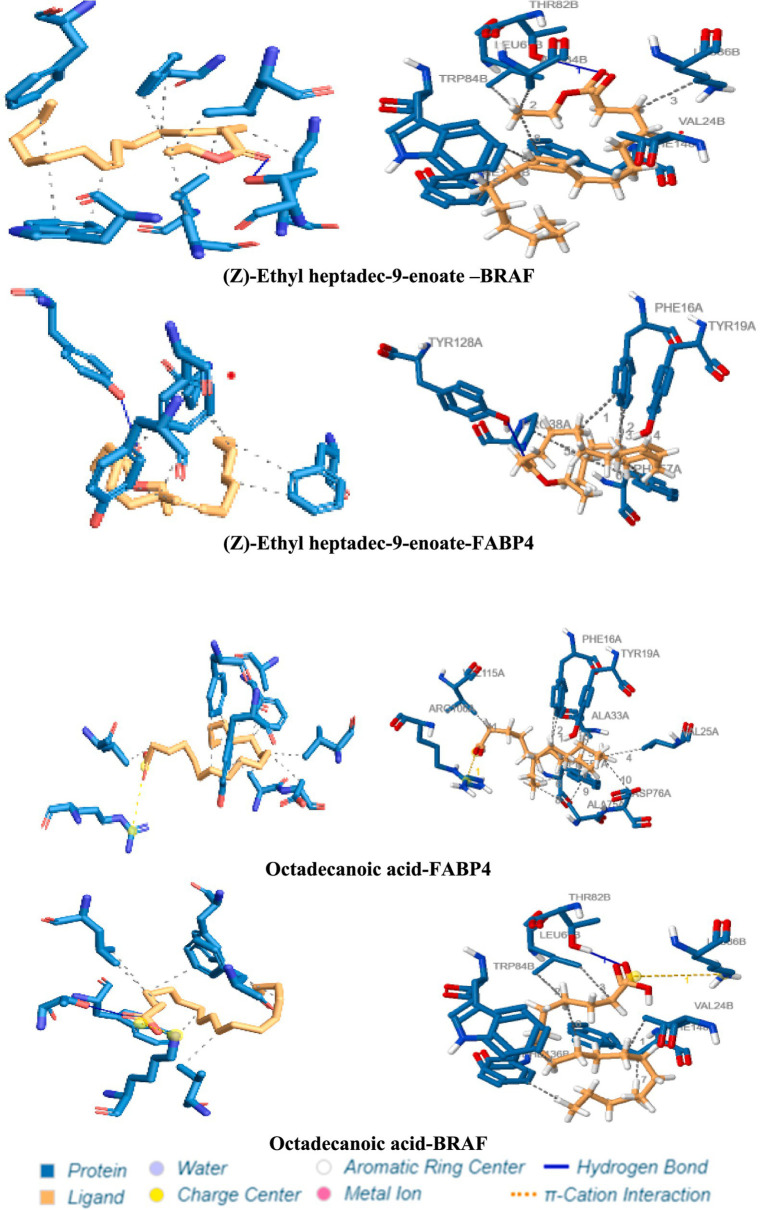
3D structure of protein-ligands complexes.

Figure 13 and Table 12 show that the interaction analysis displays that the binding of (Z)-ethyl heptadec-9-enoate to both BRAF and FABP4 was basically made through hydrophobic contacts with only a few stabilizing hydrogen bonds. One of the 3D structures of (Z)-ethyl heptadec-9- enoate-BRAF shows that the ligand formed more than one hydrophobic contacts with some important residues such as Val24, Ala34, Lys36, Leu67, Trp84, Phe136, and Phe148, the distance between the atoms involved in the interaction was approximately 3.45 to 3.90, which is a good fit of the ligand into a hydrophobic pocket of the BRAF. It makes sense that Trp84 and Phe148 interact with the ligand at least twice, as it is very probable that their role is important in binding the ligand. Apart from hydrophobic interactions, the ligand and Thr82 form a single but properly oriented hydrogen bond; the distance between hydrogen and acceptor is 2.66, between donor and acceptor 3.60, and the donor angle is 171.66, indicating a very strong interaction that contributes to the complex stability. Also, in the (Z)-ethyl heptadec-9-enoate-FABP4 complex, the ligand is mainly stabilized through extensive hydrophobic interactions involving aromatic and non-polar residues such as Phe16, Tyr19, Pro38, and Phe57, with distances between 3.61 and 3.82, consistent with the hydrophobic nature of the FABP4 binding cavity. One H bond is also with Tyr128, having a hydrogen acceptor distance of 2.44. The table also displays the pharmacophore information for octadecanoic acid in the complex of two different proteins: FABP4 and BRAF. In the case of octadecanoic acid as a ligand binding to FABP4, octadecanoic acid creates several hydrophobic interactions prior to binding with PHE16A, TYR19A, VAL25A, and ALA33A. These hydrophobic interactions occur over the distance range of 3.50 to 3.89 Å. On the other hand, in the case of octadecanoic acid as a ligand binding to the BRAF protein, there are several hydrophobic interactions, including VAL24B, LEU67B, TRP84B, PHE136B, and PHE148B; these interactions occurred over the distance of 3.45 to 3.96 Å. There was also, in this case, a single hydrogen bond between octadecanoic acid and BRAF (THR82B) that has an H–A (hydrogen to acceptor) distance of 2.08 Å, D–A (donor to acceptor) distance of 3.01 Å, and an angle of 165.28° for the donor.

**Table 12:** Pharmacophoric parameters of protein-ligand complexes.

Complexes	Hydrophobic interactions						Hydrogen bond							
	Index	Residues	AA	Distance	Ligand Atom	Protein Atom	Index	Residue	AA	Distance H-A	Distance D-A	Donor angle	Donor atom	Acceptor atom
(Z)-Ethyl heptadec-9-enoate -BRAF	1	24B	VAL	3.45	2,581	215	1	82B	THR	2.66	3.60	171.66	774 [O3]	2,585 [O2]
	2	34B	ALA	3.89	2,588	316	-	-	-	-	-	-	-	-
	3	36B	LYS	3.68	2,582	332	-	-	-	-	-	-	-	-
	4	67B	LEU	3.74	2,588	643	-	-	-	-	-	-	-	-
	5	84B	TRP	3.72	2,573	803								
	6	84B	TRP	3.57	2,571	802								
	7	136B	PHE	3.74	2,573	1,324								
	8	148B	PHE	3.83	2,588	1,439								
	9	148B	PHE	3.90	2,579	1,434								
(Z)-Ethyl heptadec-9-enoate –FABP4	1	16A	PHE	3.72	1,277	150	1	128A	TYR	2.44	2.86	106.11	1,229 [O3]	1,282 [O2]
	2	16A	PHE	3.62	1,272	151	-	-	-	-	-	-	-	-
	3	16A	PHE	3.61	1,266	152	-	-	-	-	-	-	-	-
	4	19A	TYR	3.75	1,267	181	-	-	-	-	-	-	-	-
	5	38A	PRO	3.82	1,274	353	-	-	-	-	-	-	-	-
	6	57A	PHE	3.77	1,274	524	-	-	-	-	-	-	-	-
	7	57A	PHE	3.66	1,271	526	-	-	-	-	-	-	-	-
Octadecanoic_acid-FABP4	1	16A	PHE	3.66	1,272	152								
	2	16A	PHE	3.77	1,276	150								
	3	19A	TYR	3.65	1,266	181								
	4	25A	VAL	3.54	1,268	237								
	5	33A	ALA	3.74	1,270	313								
	6	57A	PHE	3.89	1,275	524								
	7	57A	PHE	3.50	1,270	526								
	8	75A	ALA	3.73	1,275	698								
	9	75A	ALA	3.71	1,269	698								
	10	76A	ASP	3.80	1,268	704								
	11	115A	VAL	3.50	1,281	1,103								
Octadecanoic_acid -BRAF	1	24B	VAL	3.50	2,571	214	1	82B	THR	2.08	3.01	165.28	774 [O3]	2,586 [O.co2]
	2	67B	LEU	3.83	2,582	643	-	-	-	-	-	-	-	-
	3	67B	LEU	3.96	2,584	644	-	-	-	-	-	-	-	-
	4	84B	TRP	3.77	2,568	803	-	-	-	-	-	-	-	-
	5	136B	PHE	3.62	2,578	1,324								
	6	148B	PHE	3.53	2,582	1,439								
	7	148B	PHE	3.45	2,575	1,434								

### 3.8 Acute toxicity profile of *Lactaplantibacillus plantarum* compounds

Tables 13–15 present the acute toxicity profile of *L. plantarum* metabolites. The in silico acute toxicity assessment reveals significant differences in the compounds’ safety profiles. 2,4-Decadienal is predicted to be toxic through acute inhalation (52% confidence), eye irritation, and corrosion (58% confidence), thus it exhibits a less favorable toxicity profile. Besides, it is predicted as non-toxic for acute oral toxicity (83%) and acute dermal toxicity (92%). These findings indicate that the compound may cause local toxicity, especially through inhalation and ocular contact. (Z)-ethyl heptadec-9-enoate is predicted to be non-toxic for all endpoints evaluated, including acute inhalation, oral, dermal toxicity, and eye irritation, with confidence values from 56 to 100% that reflect the high reliability of these predictions. Likewise, the safety profile of the third compound is even more solid, as non-toxic is predicted for all acute toxicity endpoints with very high confidence levels (90, 100%), thus confirming the favorable toxicological profile of the compound.

**Table 13:** Acute toxicity profile of 2,4-decadienal.

Endpoint	Prediction	Confidence	Applicability domain (AD)	Predicted fragment contribution
Acute inhalation toxicity	Toxic (+)	52.0%	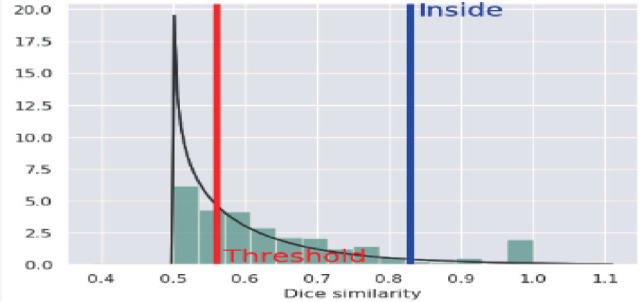	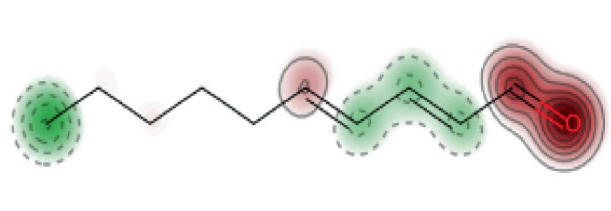
Acute oral toxicity	Non-toxic (−)	83.0%	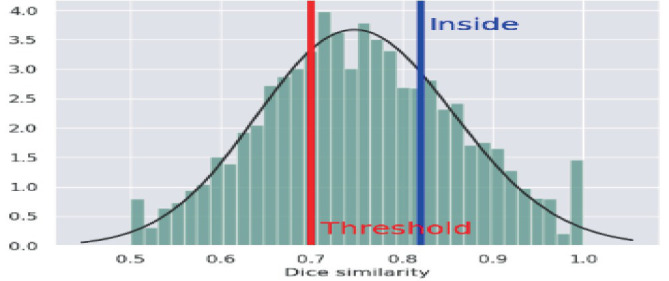	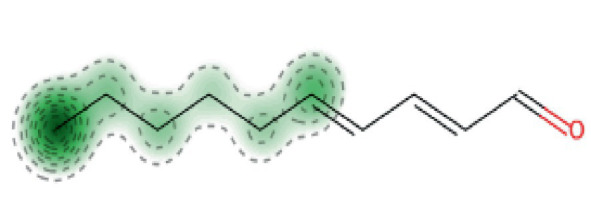
Eye irritation and corrosion	Toxic (+)	58.0%	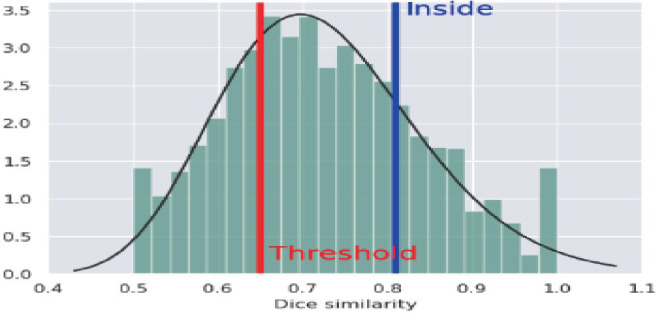	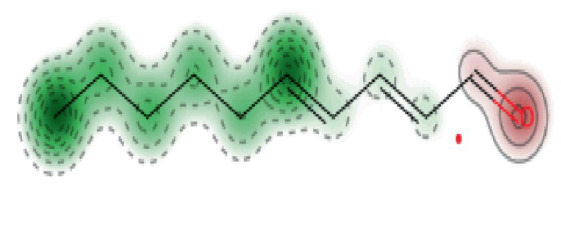
Acute dermal toxicity	Non-toxic (−)	92.0%	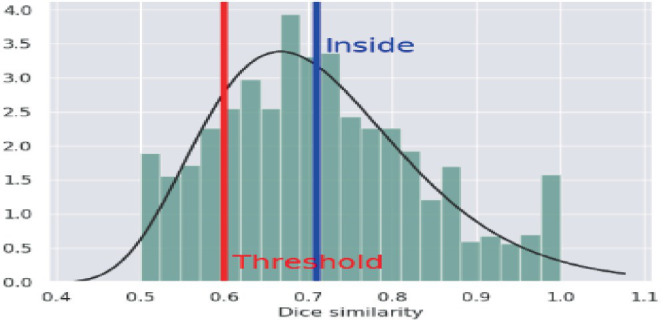	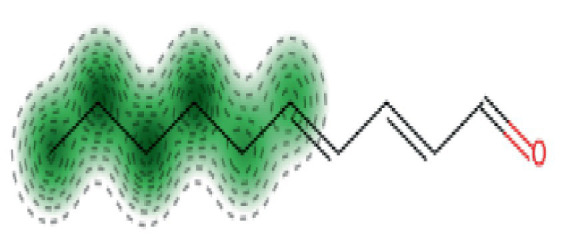

**Table 14:** Acute toxicity profile of (Z)-ethyl heptadec-9-enoate.

Endpoint	Prediction	Confidence	Applicability domain (AD)	Predicted fragment contribution
Acute inhalation toxicity	Non-toxic (+)	56.0%	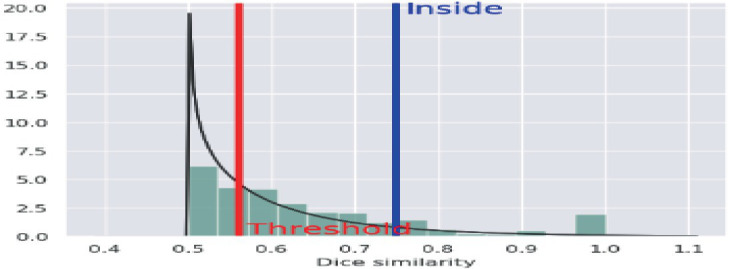	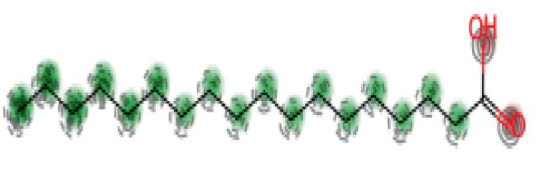
Acute oral toxicity	Non-toxic (−)	100.0%	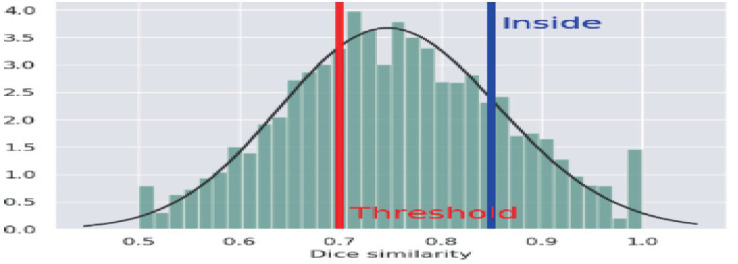	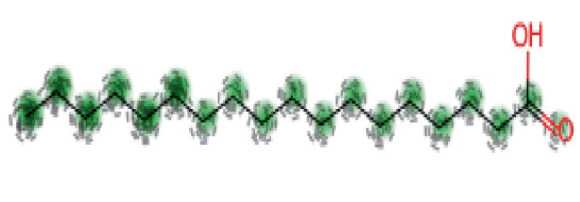
Eye irritation and corrosion	Non-toxic (−)	84.0%	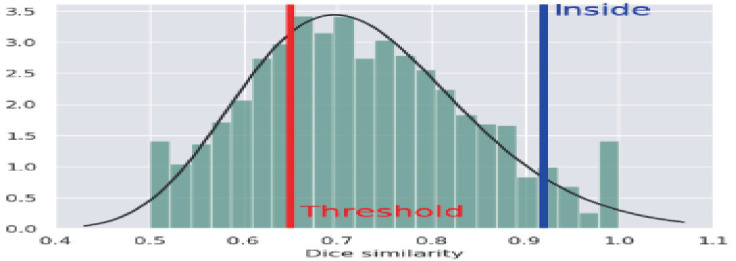	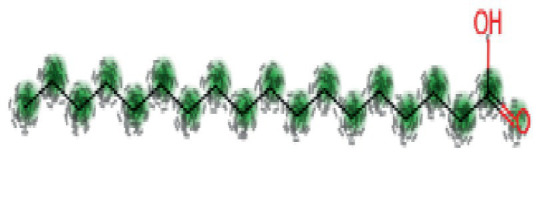
Acute dermal toxicity	Non-toxic (−)	99.0%	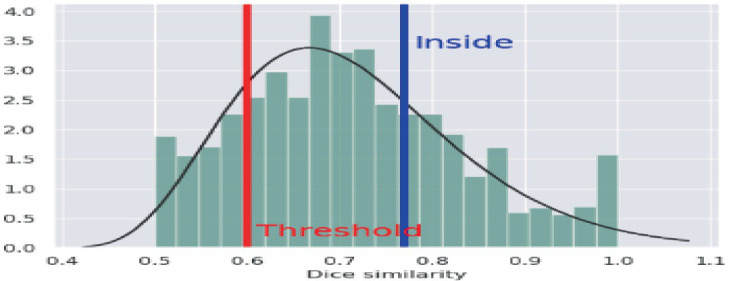	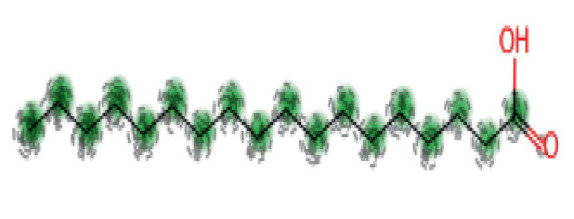

**Table 15:** Acute toxicity profile of octadecanoic acid.

Endpoint	Prediction	Confidence	Applicability domain (AD)	Predicted fragment contribution
Acute inhalation toxicity	Non-toxic (+)	90.0%	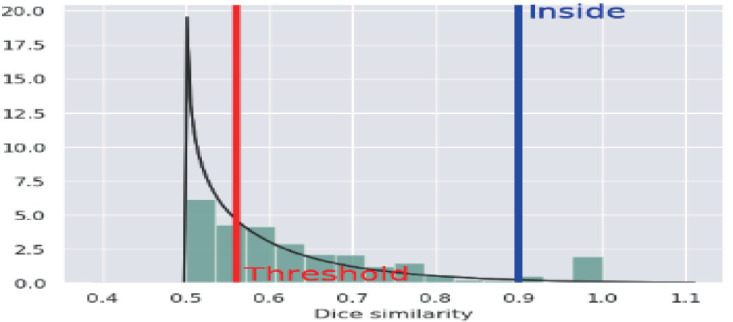	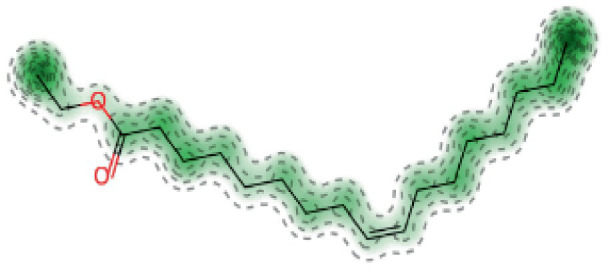
Acute oral toxicity	Non-toxic (−)	100.0%	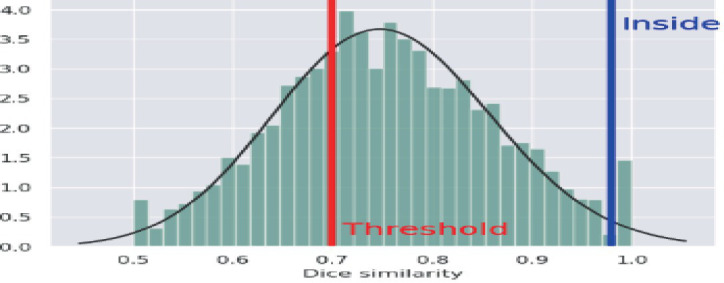	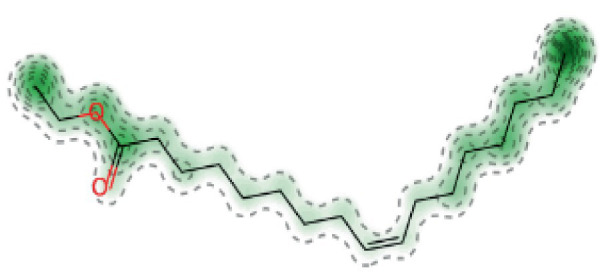
Eye irritation and corrosion	Non-toxic (−)	95.0%	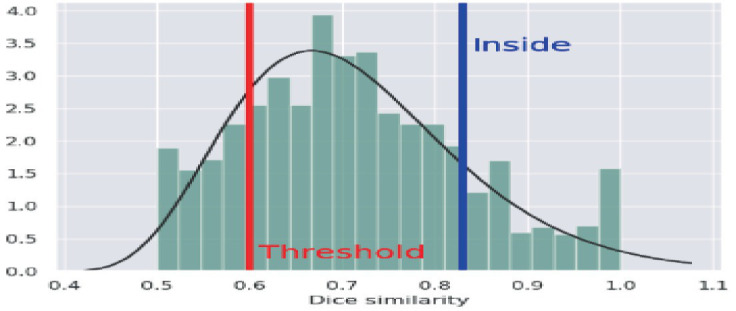	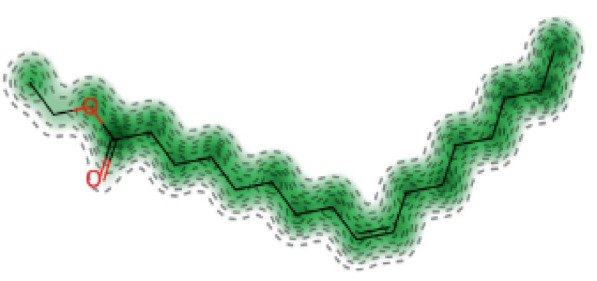
Acute dermal toxicity	Non-toxic (−)	100.0%	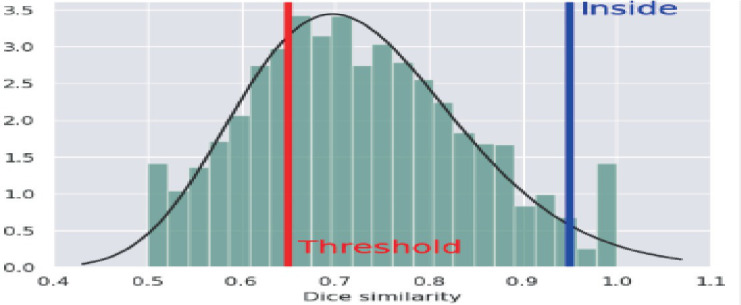	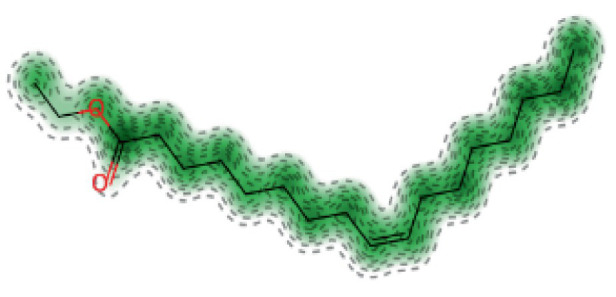

### 3.9 Dynamic simulation results

#### 3.9.1 RMSD, RMSF, and protein-ligand contacts of BRAF bind to *Lactaplantibacillus plantarum* compounds

**Figure 14:**
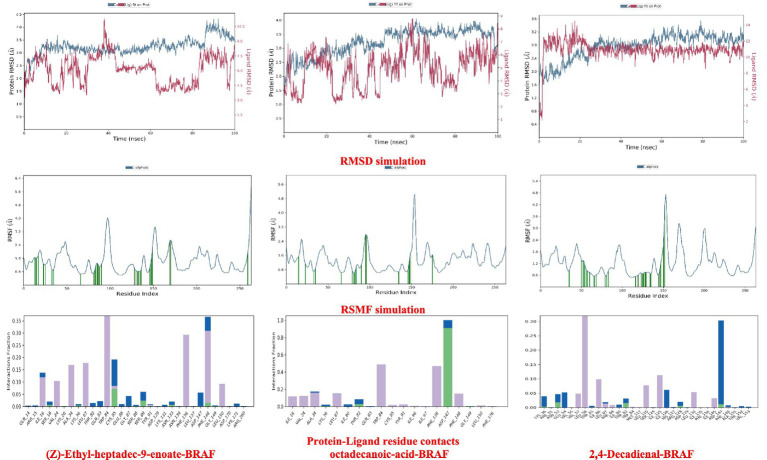
RMSD, RMSF, and protein-ligand contacts of BRAF bind to *L. plantarum* compound.

Figure 14 presents the RMSD, RMSF, and protein-ligand contacts variation during a 100 ns dynamic simulation. Based on RMSD analysis of the (Z)-Ethyl-heptadec-9-enoate–BRAF protein complex, there appears to be an initial equilibration period in the first nanoseconds of simulation; after which, the trajectory for (Z)-Ethyl-heptadec-9-enoate was stable throughout the entire simulation despite moderate variations in both protein backbone and ligand coordinate. Further, RMSD values indicated that the ligand remained stable relative to the protein; therefore, the (Z)-Ethyl-heptadec-9-enoate compound retained its overall orientation in the BRAF receptor binding pocket. The RMSF analysis supports these findings, as only a limited number of three-dimensional (3D) residues showed high flexibility throughout the protein system, whereas most of the protein residues exhibited little to no variation, with only a few residues associated with significant variations from baseline, such as those located in loop regions that are not near the catalytic site. Contact analyses between (Z)-Ethyl-heptadec-9-enoate and the BRAF demonstrated that the compound maintained contact with the receptor’s amino-acid residues throughout the simulation, predominantly via hydrophobic interactions and sporadically through hydrogen bonding; ultimately, this indicates stable binding of (Z)-Ethyl-heptadec-9-enoate with BRAF through the molecular dynamics simulation.

The root mean square deviation (RMSD) trajectory of the octadecanoic acid–BRAF complex exhibited more significant oscillations than those observed for the other complexes, which is indicative of moderate conformational reorganization of the protein backbone over time during the simulation period. While these large deviations occurred, the protein showed a relatively stable plateau between the middle of the simulation and the end of the simulation. Conversely, the RMSD of the ligand demonstrated several repositioning events regarding its position in the active site during the simulation. The root mean square fluctuation (RMSF) profile presented data for individual residues that exhibited flexibility above their average values (with the highest levels of flexibility observed in the central region of the protein), indicating that there were more localized dynamic motions taking place at these specific residues. Protein–ligand contact analysis showed only a small number of specific residues that had high interaction frequencies, while one residue had a particularly high proportion of dominant interaction fraction throughout the entire simulation. These results demonstrate that octadecanoic acid was persistently bound to the binding pocket of BRAF while undergoing relatively substantial dynamic repositioning during the entire simulation.

The complex between 2,4-Decadienal and BRAF stabilized rapidly after the initial stages of the simulation, with the RMSD analysis of both the protein and ligand largely coinciding (that is, RMSD remained relatively stable for both ligand and protein through the end of the simulation). In addition, the RMSD of the ligand tracked closely with the RMSD of the protein, indicating that the ligand was securely and stably accommodated within the receptor cavity. RMSF analysis indicated relatively low fluctuations in the majority of the complexes’ residues, with the exception of a limited number of peaks that corresponded to flexible loops; therefore, the overall structure of the protein/ligand complex was rigid. Contact analysis between the protein and ligand revealed repeated contact from several residues, with most characterized by moderate hydrophobic interactions. Collectively, these results demonstrate that 2,4-Decadienal formed a stable and long-lived (or persistent) complex to BRAF and experienced little to no change in overall structure throughout the course of the molecular dynamics simulation.

#### 3.9.2 Principal component analysis of *Lactaplantibacillus plantarum* compounds-BRAF complexes

**Figure 15:**
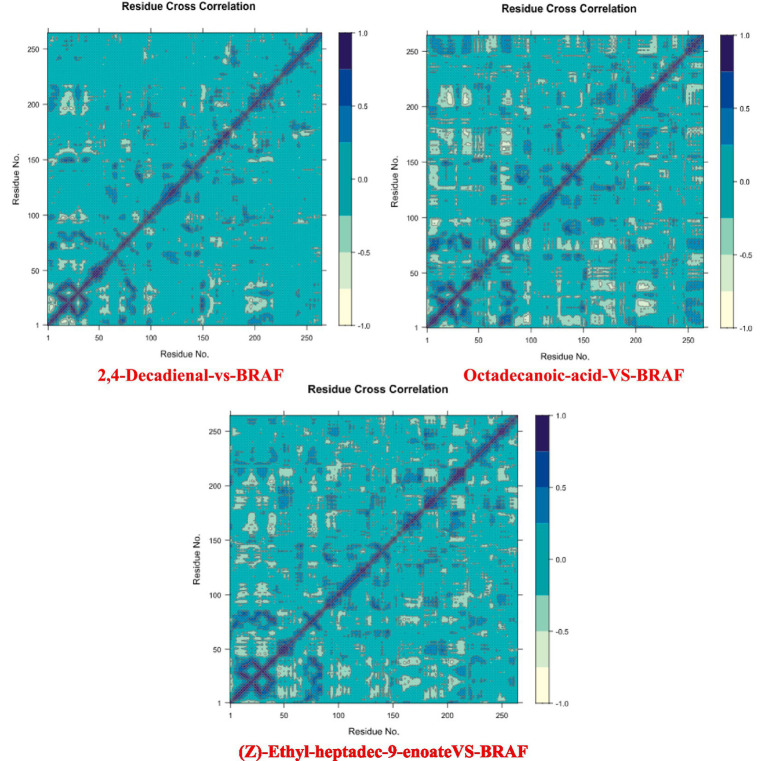
Principal component analysis of BRAF-ligands complexes.

Figure 15 shows the principal component analysis of BRAF-Ligand complexes during the dynamic simulation. According to these results, the cross-correlation matrix of the 2,4-Decadienal–BRAF complex displayed, for the majority of residues, moderately correlated motions throughout the proteins as a whole, and localized portions of the proteins displayed anti-correlated residue motions. The predominant distribution of strong positive correlations resided primarily in the diagonal region, indicative of residue movements that were highly correlated to each other based on their proximity. Only a few pairs of well-separated residues demonstrated correlated dynamics based upon long-range communication between residues in the protein during the binding of the ligand. As for the octadecanoic acid–BRAF complex, the cross-correlations demonstrate a dynamic profile that was much more heterogeneous than the 2,4-Decadienal–BRAF complex, containing both aggregated and dispersed patterns of correlated and anti-correlated residue motions distributed throughout most of the protein regions. There were more overall clusters of positive and negative correlations that were observed away from and near to the diagonal axis of the matrix, signifying the repair of cooperative movement between distant segments of residues within the protein during the binding of the ligand. Moreover, the broader distribution of both positive and negative correlations indicated an increase in dynamic coupling and conformational flexibility within the protein structure during simulation. Residue cross-correlation analysis of the (Z)-Ethyl-heptadec-9-enoate-BRAF complex indicated a pattern of strong group correlations, but smaller-scale anti-correlation areas. The presence of a dominant positive correlation along the diagonal indicated a stable coordination of adjacent residues moving in the same direction. However, off-diagonal fluctuations were generally lower, indicating that there was not much disruption to the long-range structure of the complex. In addition, the correlation map showed much more homogeneous distribution, indicating that residue movements were more constrained with a corresponding increase in dynamic stability for the protein-ligand complex.

#### 3.9.3 Principal component analysis and essential dynamics of compound–BRAF complexes

**Figure 16:**
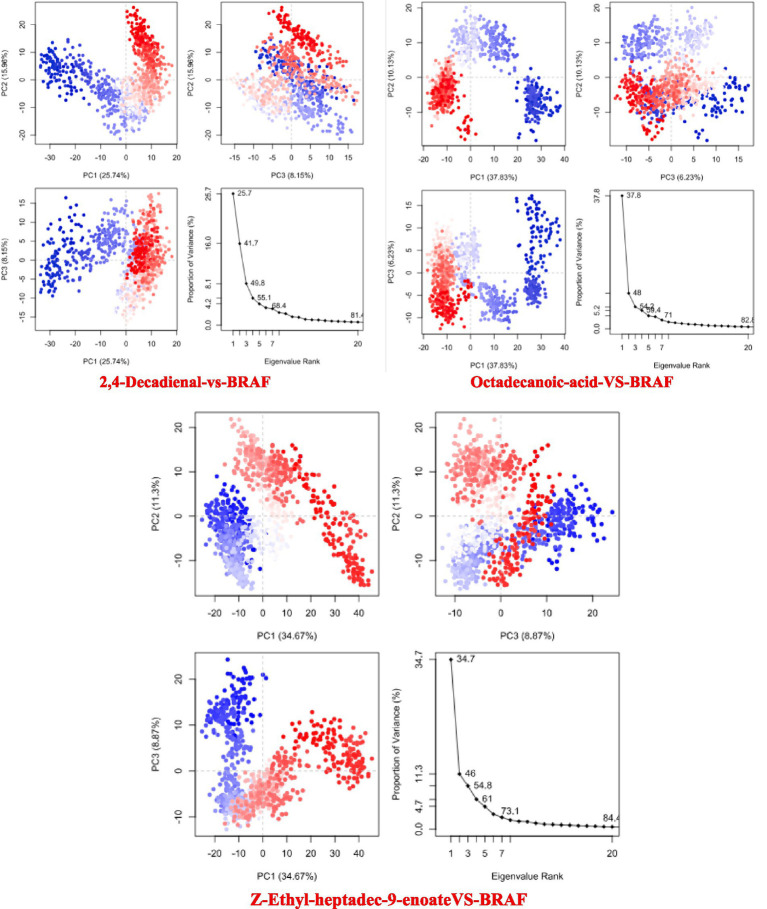
Principal component analysis and essential dynamics of compound–BRAF complexes.

Figure 16 shows the principal component analysis and essential dynamics of phytoconstituent–BRAF complexes. The 2,4-Decadienal-BRAF complex shows a large amount of conformational freedom based on considerable amounts of collective motion captured in the MD simulations, as evidenced by the trajectory cluster’s variability along the first principal component (PC1). Furthermore, the apparent separate clustering of the trajectories present in PC1 and PC2 indicates that the different conformations were vastly different from one another and were sampled over the simulation time. Each eigenvalue decay plot also shows an overall decrease in each eigenvector’s contribution to variance from the next-highest-order eigenvector. Thus, the complex has a moderate amount of conformational flexibility while still maintaining a consistent dynamic behavior over the course of the simulation. The PCA of the octadecanoic acid-BRAF complex shows the greatest amount of dispersion, indicating a significant amount of atomic movement and overall dynamic alterations. The distribution of the trajectories based on the principal component graph demonstrates significant structural alteration occurred throughout the simulation. The first eigenvalue contributes significantly to the total motion in the eigenvalue decay spectrum, while subsequent eigenvalues contribute much, much less than the first eigenvalue. The BRAF–octadecanoic acid complex has shown more flexibility and conformational variability than all other complexes during the molecular dynamics simulation.

(Z)-Ethyl-heptadec-9-enoate-BRAF recreated a tight cluster of conformations due to the significant amount of similarities in each frame along the first principal component. Therefore, (Z)-Ethyl-heptadec-9-enoate-BRAF displays lower structural volatility and thus possesses a greater level of conformational stability. There were more evenly distributed groups of trajectory frames with less frequent transitions between different conformational states throughout the simulation. A quick decrease in the eigenvalue distribution after the 1st few eigenvalues also indicates that most of the collective motion originates from a smaller number of principal modes. Together, these results support the conclusion that (Z)-Ethyl-heptadec-9-enoate-BRAF exhibits a greater level of stable dynamic behavior and a lower amount of variability of conformation throughout the entire time course of the simulation.

#### 3.9.4 RMSD, RMSF, rGyr, MolSA, and PSA of ligand–BRAF complexes fluctuation during dynamic simulation

**Figure 17:**
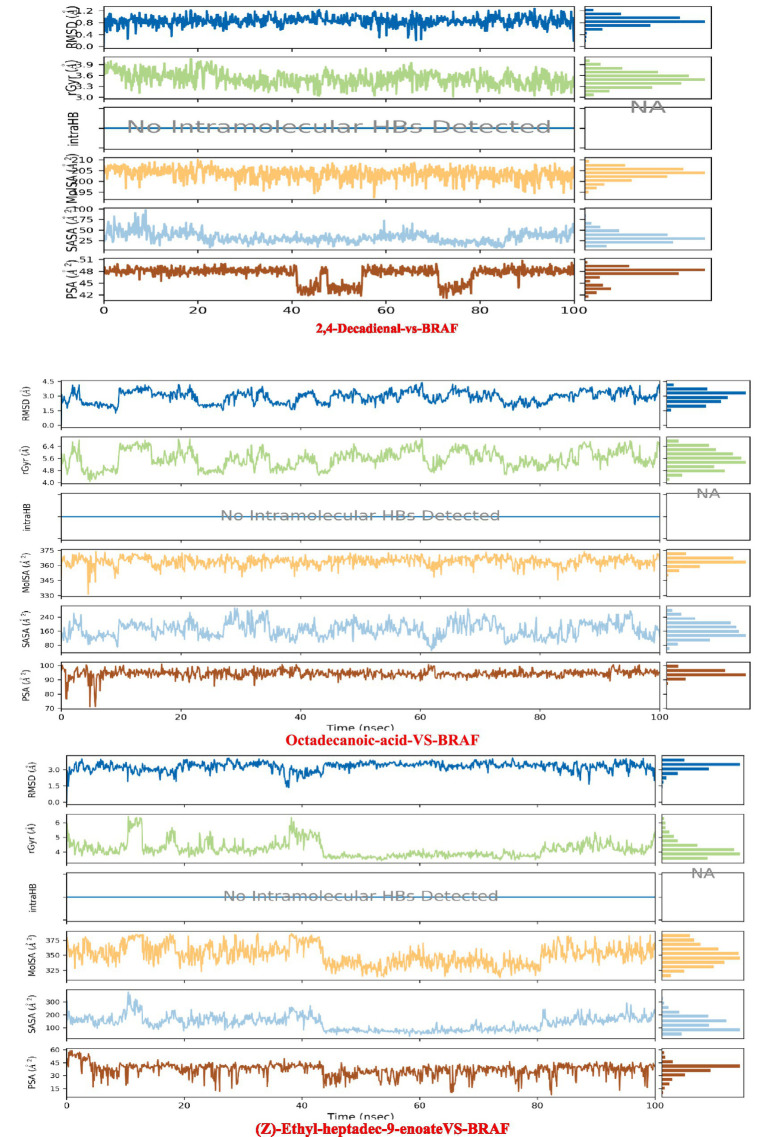
RMSD, RMSF, *r*Gyr, MolSA, and PSA of ligand–BRAF complexes fluctuation.

Figure 17 presents the RMSD, RMSF, rGyr, MolSA, and PSA of ligand–BRAF Complexes fluctuation during dynamic simulation. The MD trajectories of the complex formed between 2,4-decadienal and BRAF provide evidence that there is a stable protein-ligand association throughout the entire simulation. The RMSD of the backbone of the protein did not vary significantly (between 2.6–2.9 Å) following an initial equilibration period, indicating that the receptor had a conserved global conformational stability. The ligand RMSD had some moderate fluctuations, indicating that the ligand maintained a stable orientation in the binding site while experiencing local rearrangements. The radius of gyration (rGyr) profile showed small oscillations, which indicate that the ligand maintained compact conformations throughout the simulation. No intramolecular hydrogen bonds were formed during the trajectory, indicating that ligand stability was primarily due to hydrogen bonds formed between the ligand and receptor rather than intramolecular hydrogen bond stabilization. The molecular surface area (MolSA), solvent accessible surface area (SASA), and polar surface area (PSA) were approximately constant, supporting the notion that no large conformational changes occurred during the simulation or any solvent-induced unfolding of the complex occurred. Additionally, the interaction histogram demonstrates a stable and persistent interaction between the ligand and VS-BRAf residue and shows limited but stable flexibility. Therefore, all the data presented indicate dynamic stability of the complex formed between 2,4-decadienal and VS-BRAF and provide evidence that the ligand is able to occupy the binding pocket of VS- BRAF in a favorable manner.

The octadecanoic acid-VS-BRAF complex showed stable dynamics throughout the simulation period. The protein RMSD rapidly reached a stable value of approximately 4.0–4.3 Å and remained stable for the duration of the entire simulation, suggesting the complex was well equilibrated. Ligand RMSD values showed moderate variations, indicating that the ligand was intermittently present in the binding cavity and only moved slightly from its original location. The radius of gyration, which is a measure of compactness of the ligand, varied very little, suggesting that octadecanoic acid retained its shape during binding. Likewise, the lack of intramolecular hydrogen bonding during the entirety of the simulation suggested that stabilization of the ligand was due mostly to hydrophobic and protein-mediated interactions. The MolSA and SASA measurements were also relatively stable throughout the simulation, but there were small fluctuations in SASA that appear to be due to transient increases in the exposure of the ligand to solvent as a result of local side chain movements in the binding site. PSA values were also low and remained constant over the duration of the simulations; this is consistent with the predominantly hydrophobic character of octadecanoic acid.

The complex formed by the ligand (Z)-ethyl-hexadec-9-enoate and VS-BRAF exhibited similar stable MD behavior as did the other complexes of VS-BRAF during the simulation. Protein backbone RMSD showed little fluctuation throughout the simulation, remaining between ~4.0–4.3 Å, indicating that the receptor maintained its structural stability following equilibration. Ligand RMSD also remained very low and displayed periodic fluctuations during the simulation, indicating stable ligand binding and somewhat constrained conformational flexibility in the active site. Radius of gyration (Rg) profiles were also consistent throughout the simulation, indicating that the ligand was maintained in a compact manner throughout the length of the MD simulation. As was the case for the other complexes, there were no intramolecular hydrogen bonds for the complex formed between (Z)-ethyl-hexadec-9-enoate and VS-BRAF, which indicates that the ligand primarily maintained its stability owing to its intermolecular interactions with the residues comprising VS-BRAF. Both MolSA and SASA of the complex showed slight to moderate fluctuations during the MD simulation, indicating modest changes to the solvent exposure of the ligand without any effects on the overall stability of the binding of the ligand to VS-BRAF. In addition, the PSA of the ligand was consistently low throughout the MD simulation, consistent with its hydrophobicity and likely contributing to its insertion into the hydrophobic regions of the VS-BRAF binding pocket.

## 4 Discussion

Intestinal dysbiosis, an imbalance of gut microbial composition and function, is increasingly being understood as a central factor causing metabolic, inflammatory, and immune-mediated disorders (1). A state of imbalance like this breaks down host microbiota homeostasis, resulting in changes in lipid metabolism, chronic low-grade inflammation, epithelial barrier dysfunction, and abnormal communication of host molecular pathways through signaling (47). Furthermore, microbial metabolites have been identified as crucial mediators of host-microbe communication, capable of regulating host proteins involved in metabolism, inflammation, and cellular stress response (48). Among probiotic species, *Lactaplantibacillus plantarum* has been identified as a major source of bioactive lipid-derived metabolites that can exert potential regulatory effects on the host (49, 50). The present investigation was designed to examine, using an integrated *in silico* multi-scale approach, the therapeutic potential of *L. plantarum*, derived metabolites 2,4-decadienal, (Z)-ethyl heptadec-9-enoate, and octadecanoic acid in the setting of intestinal dysbiosis. The primary aim was to uncover how these molecules can affect the molecular targets associated with dysbiosis by (i) showing good ADMET and physicochemical properties, (ii) directly interacting with key proteins involved in dysbiosis-related signaling pathways, and (iii) stabilizing these interactions through an energetically favorable binding and dynamic stability. Particular attention was given to BRAF and FABP4, which emerged as central nodes in the network pharmacology analysis and are mechanistically linked to lipid metabolism, inflammatory signaling, epithelial integrity, and stress responses in the gut.

*Lactaplantibacillus plantarum* metabolites derived from the ADMET and physicochemical profiling showed pharmacokinetic behaviors of the different components that complemented one another and were very relevant to the intestinal targeting. All the compounds had molecular weights ≤ 300 g/mol, low topological polar surface area (TPSA < 30), and no aromatic rings, thus pointing to structural simplicity and metabolic accessibility (51). Such characteristics are usually related to the compound’s ability to permeate efficiently through the membrane and to the less steric hindrance in protein binding sites. The elevated fraction of sp. carbons that were detected for (Z)-ethyl heptadec-9-enoate (0.84). Lipophilicity appeared to be the main characteristic that separates one compound from another. 2,4-Decadienal, for instance, showed a moderate level of lipophilicity (consensus LogP = 2.85); thus, the permeability through the membrane was caused to be balanced by its solubility in water, whereas (Z)-ethyl heptadec-9-enoate and octadecanoic acid had significantly high lipophilicity (consensus LogP = 6.01). Such high lipophilicity is typical for fatty acid-like molecules and thus explains the binding with lipid-associated targets (52). On the one hand, the high lipophilicity may hinder solubility, but on the other hand, this characteristic is beneficial in the intestinal environment, where the lipid transport proteins and micellar systems take care of the absorption.

Pharmacokinetic predictions supported that all compounds would be highly absorbed through the gastrointestinal tract, making them suitable for oral administration and local action in the gut (53). Notably, 2,4-decadienal was predicted to be capable of crossing the blood–brain barrier, while (Z)-ethyl heptadec-9-enoate and octadecanoic acid were predicted to lack this ability. This indicates a lower risk of the central nervous system exposure for these two compounds, which is a major safety consideration in the long-term modulation of gut pathways. Additionally, none of the compounds were predicted to be substrates of P-glycoprotein, indicating that there would be limited efflux and possibly increased intestinal retention (54). Altogether, these ADMET properties are indicative of the metabolites being modulators of the gut without having a systemic drug effect, which is consistent with the aim to therapeutically restore intestinal homeostasis. Furthermore, acute toxicity predictions demonstrated that the safety profiles of (Z)-ethyl heptadec-9-enoate and octadecanoic acid were very good, with the compounds predicted to be non-toxic at inhalation, oral, dermal, and ocular levels with a high degree of assurance. On the other hand, 2,4-decadienal was predicted to be potentially toxic to the lungs and eyes; its therapeutic use might involve the development of a suitable formulation or dosage control.

Fatty acid-binding protein 4 (FABP4) is essentially involved in intracellular lipid trafficking, fatty acid signaling, and inflammatory regulation (55). Originally identified as a marker of adipocytes and macrophages, FABP4 is now being linked more and more with intestinal inflammation and metabolic dysregulation. During dysbiosis, increased circulating and locally expressed FABP4 levels lead to lipid over-accumulation, triggering of the inflammatory pathways, and intensification of cytokine signaling (55). FABP4 acts in the transport of fatty acids to nuclear receptors such as PPARG, thus modifying transcriptional programs related to inflammation and metabolism (56). Molecular docking studies have indicated that fatty acid metabolites derived from *L. plantarum* can bind stably inside the FABP4 cavity. (Z)-Ethyl heptadec-9-enoate and octadecanoic acid showed binding energies of −5.8 kcal/mol, whereas 2,4-decadienal exhibited a little lower affinity (−5.1 kcal/mol). The attachment of these compounds was mainly facilitated by hydrophobic interactions with essential residues such as Phe16, Met20, Ala33, Phe57, Ala75, and Val115, which is in line with the hydrophobic characteristic of the FABP4 binding pocket. In addition, the ligand stability and orientation were further supported by hydrogen bonds between Thr79 and Arg126. On the one hand, by occupying the FABP4 binding site, these metabolites could thus competitively block the binding of endogenous fatty acids, which in turn would decrease inflammatory signaling and lipid-induced activation of PPARG-dependent pathways (57). Hence, a molecular connection exists between microbial lipid metabolites and host transcriptional regulation under dysbiosis through the direct inhibition of FABP4 by these metabolites, which is supported by the fact that FABP4 is regulated by transcription factors such as PPARG and MEOX1, both of which were identified in the network analysis (58).

FABP2 is a fatty acid-binding protein specific to the intestine and mainly localized in the membrane of enterocytes, where it is involved in the uptake and intracellular transport of dietary fatty acids (59). In a state of dysbiosis, changes in expression and function of FABP2 due to altered gut microbiota play a role in the mechanisms of maldifferentiated lipid absorption, consequent to an increase in intestinal permeability, leading to metabolic endotoxemia (60). Increased levels of FABP2 have been correlated with epithelial injury and inflammatory bowel diseases, and thus, it represents a pathophysiological marker and latent therapeutic target in conditions associated with dysbiosis (61). Even though the main purpose of this work was not to study FABP2 docking results, the high lipophilic character and structural similarity to fatty acids of (Z) ethyl heptadec-9-enoate and octadecanoic acid intrinsically imply that both compounds have a great probability of interacting with FABP2. Through network pharmacology, FABP2 was identified as a common target regulated by intestinal transcription factors such as CDX1, CDX2, and NR5A2, which are crucial for gut epithelial identity and lipid handling (62, 63). Decreasing the flux of lipids through the enterocytes by controlling FABP2 with these metabolites might be one way to limit lipid-induced inflammation and to restore the epithelial barrier function.

BRAF is a MAPK/ERK pathway serine/threonine kinase, mainly known for oncogenic signaling and cell proliferation (64). But, recent studies are showing that aberrant MAPK signaling is involved in intestinal inflammation, epithelial stress responses, and immune dysregulation as well (65). Along with dysbiosis, a microbial imbalance can cause a continuous activation of MAPK pathways, which results in excessive epithelial turnover, production of inflammatory cytokines, and compromised barrier integrity (66). Docking analysis showed that (Z)-ethyl heptadec-9-enoate, octadecanoic acid, and 2,4-decadienal are able to bind well to the BRAF, with their binding energies ranging from −5.5 to −6.2 kcal/mol, thus being on par with the reference inhibitor vemurafenib (−5.9 kcal/mol). These were mainly hydrophobic interactions, involving amino acid residues such as Trp84, Val24, Ala34, Leu67, Phe136, as well as Phe148, with some additional stabilization by alkyl interactions. Most (Z)-ethyl heptadec-9-enoate had the greatest propensity for binding (−6.2 kcal/mol), thereby pointing to a successful occupancy of the kinase’s active site. The molecular normal mode analysis of the (Z)-ethyl heptadec-9-enoate-BRAF complex corroborated the low deformability, B-factor constancy, low eigenvalue, and rigidity pattern encompassing the active site, thus confirming the stability of the complex. By blocking BRAF, these metabolites could prevent too much MAPK activation in the intestinal lining, which would then lead to less inflammatory signaling cascades and the promotion of epithelial homeostasis (67). FKBP5 is a protein that assists the chaperones and plays a role in glucocorticoid receptor regulation and stress-responsive signaling (68). Changes in the functioning of FKBP5 have been associated with chronic inflammation, altered immune responses, and stress-related gastrointestinal disorders. As for the gut, FKBP5, through modulating the inflammatory sensitivity and epithelial responses to stress hormones, might be implicated in the mechanism of dysbiosis-associated pathology (69). Network analysis pointed out FKBP5 as a common target regulated by transcription factors such as ATOH8, thus implicating it in the dysbiosis regulatory network. Even if there are no direct docking results for FKBP5, metabolites of *L. plantarum* have the potential to affect the expression and activity of FKBP5 indirectly by changing the kinases and transcription factors that are upstream.

The findings of the molecular dynamics simulation of the phytoconstituent-VS-BRAF complexes exhibit favorable structural stability and a consistent presence of ligand-protein interactions throughout the simulated time period. This supports the validity of the docking results. The relatively constant RMSD values of the protein backbones indicate that ligand binding did not significantly destabilize the conformation of the receptor, while moderate RMSD fluctuations of the ligands help show that the ligands can adapt or readapt into the active site. Such results from molecular dynamics studies have been associated previously with stable inhibitor binding and maintaining receptor integrity. Furthermore, the minor differences in radius of gyration, MolSA, SASA, and PSA values indicate that the ligands maintained compact conformations and stable solvent-exposure during the simulated time frames. The absence of intramolecular hydrogen bonds for the complexes suggests that the intermolecular hydrogen bond interactions, specifically hydrophobic interactions and residue stabilization, were the main mechanisms by which the ligand was retained at the VS-BRAF binding pocket. Together, the above evidence indicates that the analyzed compounds have the potential to inhibit VS-BRAF via stable, energetically favorable ligand-receptor interactions.

## 5 Conclusion

The aim of this study was to evaluate the anti-intestinal dysbiosis effects of *L. plantarum* compounds. GC–MS analysis revealed the presence of (Z)-ethyl heptadec-9-enoate, octadecanoic acid, and 2,4-Decadienal, whose anti-intestinal dysbiosis properties are not documented in the literature. The ADME analysis showed that the compounds studied were all able to cross the intestinal barrier and had moderate fat solubility. Network pharmacology indicated that the compounds ((Z)-ethyl heptadec-9-enoate, octadecanoic acid, and 2,4-Decadienal) could bind to the target proteins BRAF and FABP4, which are involved in intestinal dysbiosis, and could interfere with signaling pathways such as the regulation of the MAPK cascade, the cell surface receptor protein tyrosine kinase signaling pathway, and biological processes such as the response to bacteria and intestinal absorption. Molecular docking indicated that the (Z)-ethyl heptadec-9-enoate, octadecanoic acid, and 2,4-Decadienal could bind to the BRAF and FABP4 proteins with a greater affinity those of the reference treatment (Rifaximin) for intestinal dysbiosis. These interactions were confirmed by a pharmacophore analysis and were predominantly due to hydrophobic interactions. Dynamic simulation indicates that compounds have the potential to inhibit VS-BRAF via stable, energetically favorable ligand-protein interactions. Prediction of the acute toxicity of these compounds indicated that they exhibited no toxicity via oral, dermal, or inhalation routes. This toxicity profile indicates that these compounds are safe for use as agents against intestinal dysbiosis. Despite these numerous findings, this study remains entirely predictive and computational in nature and, therefore, requires experimental validation to confirm the predictive data.

## Data Availability

The original contributions presented in the study are included in the article, further inquiries can be directed to the corresponding authors.

## Footnotes

1
https://pubchem.ncbi.nlm.nih.gov/

2
https://www.swissadme.ch/index.php

3
https://www.swisstargetprediction.ch/predict.php

4
https://www.genecards.org/

5
https://www.interactivenn.net/

6
https://string-db.org/

7
https://metascape.org/

8
https://www.bioinformatics.com.cn/login_en/

9
https://saves.mbi.ucla.edu/

10
https://saves.mbi.ucla.edu/

11
https://mcule.com/apps/1-click-docking/

12
https://imods.iqf.csic.es/

13
https://plip-tool.biotec.tu-dresden.de/plip-web/plip/index

## Glossary

ADMET: Absorption, Distribution, Metabolism, Excretion, and Toxicity
TPSA: Topological Polar Surface Area
FABP: Fatty Acid–Binding Protein
FABP2: Fatty Acid–Binding Protein 2
FABP4: Fatty Acid–Binding Protein 4
BRAF: B-Raf Proto-Oncogene, Serine/Threonine Kinase
FKBP5: FK506 Binding Protein 5
PPI: Protein–Protein Interaction
CYP: Cytochrome P450
GI: Gastrointestinal
BBB: Blood–Brain Barrier
SAR: Structure–Activity Relationship
PDB: Protein Data Bank
CSNK2A1: Casein Kinase 2 Alpha 1
CDX1: Caudal Type Homeobox 1
CDX2: Caudal Type Homeobox 2
NR5A2: Nuclear Receptor Subfamily 5 Group A Member 2
MMP2: Matrix Metallopeptidase 2
SCD: Stearoyl-CoA Desaturase
APP: Amyloid Beta Precursor Protein
HDAC1: Histone Deacetylase 1
HDAC3: Histone Deacetylase 3
RPS4X: Ribosomal Protein S4, X-Linked
GSK3B: Glycogen Synthase Kinase 3 Beta
